# Disabling leading and lagging strand histone transmission results in parental histones loss and reduced cell plasticity and viability

**DOI:** 10.1126/sciadv.adr1453

**Published:** 2025-02-19

**Authors:** Leonie Kollenstart, Alva Biran, Nicolas Alcaraz, Nazaret Reverón-Gómez, Victor Solis-Mezarino, Moritz Völker-Albert, Fion Jenkinson, Valentin Flury, Anja Groth

**Affiliations:** ^1^ Novo Nordisk Foundation Center for Protein Research, University of Copenhagen, Copenhagen 2200, Denmark.; ^2^ EpiQMAx GmbH, Planegg, Germany.; ^3^ Biotech Research and Innovation Centre, University of Copenhagen, Copenhagen 2200, Denmark.; ^4^ Department of Cellular and Molecular Medicine, University of Copenhagen, Copenhagen 2200, Denmark.

## Abstract

In the process of DNA replication, the first steps in restoring the chromatin landscape involve parental histone recycling and new histone deposition. Disrupting histone recycling to either the leading or lagging strand induces asymmetric histone inheritance, affecting epigenome maintenance and cellular identity. However, the order and kinetics of these effects remain elusive. Here, we use inducible mutants to dissect the early and late consequences of impaired histone recycling. Simultaneous disruption of both leading (POLE4) and lagging strand (MCM2-2A) recycling pathways impairs the transmission of parental histones to newly synthesized DNA, releasing some parental histones to the soluble pool. Subsequently, H3K27me3 accumulates aberrantly during chromatin restoration in a manner preceding gene expression changes. Loss of histone inheritance and the ensuing chromatin restoration defects alter gene expression in embryonic stem cells and challenge differentiation programs and cell viability. Our findings demonstrate the importance of efficient transmission of histone-based information during DNA replication for maintaining chromatin landscapes, differentiation potential, and cellular viability.

## Teaser

Losing parental histones during DNA replication fork passage challenges differentiation competence and cell viability.

## INTRODUCTION

Multicellular organismal development requires the execution of specific transcriptional programs to generate a diverse array of cell types. Despite having a single genome, highly specialized cell types can be produced and maintained in part through epigenetic regulation, highlighting the remarkable capacity of cells for both plasticity and memory. The accurate maintenance of the epigenetic landscape during cell division is crucial for maintaining cell identity and proper cellular function. During cell division, the chromatin landscape is disrupted by replication fork passage. Parental histones are disassembled in front of the replication fork, moved across the replisome, and reassembled into nucleosomes on the two daughter DNA strands. In parallel, new acetylated histones are deposited to maintain nucleosome density. The recycling of parental histones with their posttranslational modifications (PTMs) is accurate, maintaining positional information, and leads to largely symmetric distribution of parental histones on leading and lagging DNA strands (*1*–*6*). Histones H3-H4 are recycled as tetramers (*7*) and/or possibly as hexamers, together with a single H2A/H2B dimer (*8*). The replisome factors Tof1/TIMELESS, minichromosome maintenance complex component 2 (MCM2), Ctf4, and DNA polymerases alpha (POLA1) and delta (POLD) are required for efficient histone recycling to lagging strand, while DNA polymerase epsilon subunits POLE3/4 facilitate recycling to the leading strand (*2*, *3*, *8*–*13*). In addition, Mrc1/CLASPIN regulates histone recycling to both daughter strands (*14*–*16*). The deposition of new histones in nascent chromatin results in a twofold dilution of prereplication histone PTM levels (*17*). To restore the prereplication chromatin states, new histones are modified in a heterogeneous process that proceeds with modification and locus-specific kinetics and, for repressive modifications, extends into the next cell cycle (*1*, *5*, *17*–*19*). In this process, parental histone PTMs can contribute to modification of new histones through read-write–based spreading (*20*, *21*), which are well documented for the repressive modifications H3K27me3 and H3K9me3 (*22*–*28*).

In an MCM2 histone–binding mutant (MCM2-2A), parental histones are recycled primarily to the leading strand, resulting in a strong asymmetry of histone PTM landscapes between sister chromatids (*2*, *3*). In mouse embryonic stem cells (mESCs), these regional asymmetries in chromatin states between sister chromatids can be inherited by daughter cells (*18*). Mutant mESCs with asymmetric histone recycling show deregulation of the chromatin landscape, primarily affecting H3K27me3 and H3K9me3, and gene expression (*18*, *19*). While H3K9me3 is lost across repeat elements, consistent with a requirement for read-write propagation in cis, H3K27me3 accumulates aberrantly (*11*, *18*, *19*) due to elevated recruitment of polycomb repressive complex 2 (PRC2) to the lagging strand (*18*). While an MCM2-2A mutant does not lose parental histones, the failure to transmit parental histones to both daughter strands challenges genome regulation and mESC identity. The cells show reduced plasticity in transitioning between embryonic stem cell states, with elevated occupancy in naïve pluripotency and two-cell–like states compared to lineage primed, and impaired embryonic differentiation (*18*).

While cis-based inheritance of histone modification is required for epigenome maintenance, there are also experimental data and modeling work supporting that the overall concentration or density of parental histones on newly synthesized DNA contributes to the restoration of modifications on the new histones (*29*–*31*). However, it remains largely unknown what happens if parental histones are lost in transmission. Moreover, it has not been characterized what happens if both leading and lagging histone recycling pathways are disabled. Here, we developed an inducible POLE4 degron system to address the initial effects of defective leading strand recycling, alone and in combination with the lagging strand recycling defect in MCM2-2A mESC. We demonstrate that deregulation of H3K27me3 (*18*, *32*) is an early consequence of asymmetric histone recycling, occurring before changes in gene expression. The combined loss of leading and lagging strand recycling resulted in loss of histone-based information during replication. This in turn aggravated gene expression changes and differentiation defects seen in cis-based inheritance mutants and challenged mESC viability.

## RESULTS

### Asymmetric histone recycling directly deregulates H3K27me3

**Fig. 1.**
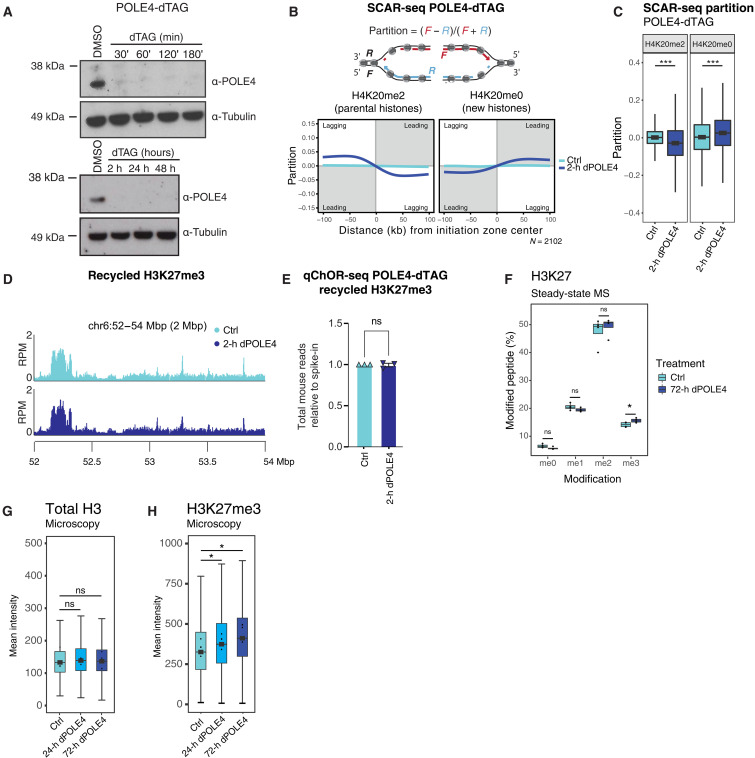
Inducing asymmetry through loss of POLE4 leads to increased global H3K27me3 levels within 24 hours. (**A**) Western blot analysis of POLE4-dTAG mESC with DMSO and dTAG-13 treatment. (**B**) Average SCAR-seq profile showing the H4K20me2 and H4K20me0 partition in 1-kb windows around initiation zones (IZs) of DMSO-treated (Ctrl) or dTAG-treated (dPOLE4) in POLE4-dTAG mESC. Partition is calculated as the proportion of forward (F) and reverse (R) read counts [(*F* − *R*)/(*F* + *R*)], see Materials and Methods. *n* = 2 biological replicates. (**C**) Box plots of H4K20me2 and H4K20me0 partition in 1-kb windows around IZ in POLE4-dTAG cells. Windows upstream of IZs were multiplied by −1. Lines indicate median, boxes represent first, and third quartiles and whiskers extend 1.5× interquartile range. Wilcoxon signed-rank test. ***P < 2.220446e-16. (**D**) ChOR-seq occupancy tracks for H3K27me3 on nascent DNA in DMSO (Ctrl, top)–or 2-hour dTAG-13 (dPOLE4, bottom)–treated POLE4-dTAG cells. Signal is quantified with reads per million (RPMs). (**E**) Bar plot of mapped reads (mouse) relative to spike-in reads (*Drosophila*) for H3K27me3 in POLE4-dTAG mESC treated with DMSO (Ctrl) or dTAG-13 (dPOLE4). Normalized to input. (**F**) Box plot showing H3K27 methylation levels quantified by MS after 72-hour dTAG-13 treatment. *n* = 4 independent replicates. Box plot as in (C). Two-sided Welch’s *t* test false discover rate (FDR). *P = 0.031. (**G** and **H**) Box plot showing total H3 (G) and H3K27me3 (H) levels quantified with high-content microscopy in POLE4-dTAG mESC-treated with DMSO (Ctrl) or dTAG-13 (dPOLE4). *n* = 3 biological replicates. Box plot as in (C). *P* values, Student *t* test (two-tailed, paired), performed on the means of the biological replicates. Ctrl vs 24-h dPOLE4 (top) *P = 0.018, Ctrl vs 72-h dPOLE4 (bottom) *P = 0.04. h, hours; ns, not significant.

To circumvent adaptation and clonal variation associated with steady-state mutants, we fused leading strand recycling factor POLE4 to a degradation tag (dTAG) (*33*) degron to establish an inducible depletion system in mESC. In mammalian cells, POLE4 knockout (KO) results in a moderate recycling bias reflecting a partial disruption of leading strand recycling, while stronger asymmetry is observed in MCM2 and POLA1 mutants defective in lagging strand recycling (*1*, *18*). In the presence of dTAG-13, POLE4 was efficiently depleted (dPOLE4) within 30 min (Fig. 1A), allowing us to investigate the immediate effects of acute histone recycling loss. We examined the relative histone occupancy on lagging and leading DNA strands, immediately after POLE4 depletion using sister chromatids after replication (SCAR)-sequencing (*3*, *34*). Short-term depletion of POLE4 resulted in moderate asymmetry of parental histones marked by H4K20me2 (*17*), biased toward the lagging strand (Fig. 1, B and C). This is consistent with observations in POLE4-KO mESCs (*1*, *11*, *35*), although the effect of POLE4 depletion is less strong, probably reflecting incomplete degradation. A comparable leading strand bias was observed for new histones marked by H4K20me0, arguing that the reduction of parental histones on the leading strand is compensated by increased deposition of new histones (Fig. 1, B and C). To address the positional accuracy and overall efficiency of histone recycling, we measured the occupancy of H3K27me3 [absent from newly synthesized histones (*17*)] in nascent chromatin using spike-in corrected chromatin occupancy after replication (qChOR)-sequencing (*34*). Unlike H4K20me2, H3K27me3 occupies specific sites across the genome, thus serving as a reliable marker for maintenance of positional information during parental histone transmission. The overall landscape and the total levels of recycled H3K27me3, including the distribution over H3K27me3 peaks and transcription start sites (TSSs), were unaltered upon removal of POLE4 (Fig. 1, D and E, and fig. S1, A and B). This argues that parental histones are rerouted from the leading strand to the lagging strand, maintaining their genomic position, as the accuracy of parental histone recycling was unaltered upon POLE4 depletion. Recycling efficiency was unaffected or even slightly increased after POLE4 depletion (fig. S1, E and F) in line with previous observations (*9*). Stable histone recycling mutants such as POLE4-KO and MCM2-2A mESCs demonstrate various changes in gene expression and repeat up-regulation (*11*, *18*, *19*). We found no detectable differences in gene or repeat expression upon 24 hours of POLE4 depletion (fig. S1G). However, alteration in gene expression became apparent after 96 hours of POLE4 depletion, overall showing a strong correlation to differentially expressed (DE) genes in POLE4-KO cells (178 genes) (*18*), although only a smaller fraction of these passed the strict cutoff for DE (29 genes) (fig. S1H and I). This argues that population-level steady-state expression changes in histone recycling mutants are driven by multiple rounds of asymmetric inheritance with associated defective chromatin maturation (*18*). To further examine the interplay of histone recycling and restoration of the PTM landscape after DNA replication, we performed mass spectrometry (MS) analysis after 72 hours of POLE4 depletion. This revealed that the levels of total histones, replicative histone H3.1 compared to the replacement variant H3.3 and new (H4K20me0) and old (H4K20me2), remained unaltered (fig. S1, J to L), consistent with the conclusion that old parental histones are rerouted but not lost upon POLE4 depletion. While H3K9me3 and H3K36me3 levels remained unaltered (fig. S1, M and N), the levels of H3K27me3 increased (Fig. 1F). Elevated H3K27me3 was previously reported in MCM2-2A histone recycling mutants (*18*, *19*, *36*), as a sign of epigenome perturbation. To resolve the kinetics of this response, we quantified H3K27me3 levels by high-content microscopy. While histone H3 levels were stable (Fig. 1G), H3K27me3 accumulated gradually after POLE4 depletion becoming significantly increased compared to wild type (WT) within 24 hours corresponding to about 2 cell cycles (Fig. 1H and fig. S1O). As POLE4 depletion did not challenge the efficiency or accuracy of H3K27me3 recycling, our data indicate that H3K27me3 establishment on new histones is deregulated, resulting in progressive aberrant accumulation of this modification during chromatin restoration in the wake of defective recycling. This demonstrates that H3K27me3 accumulation is an early response to histone recycling asymmetry preceding gene expression changes, consistent with the notion that epigenome alterations drive gene expression changes in these models (*1*, *18*, *19*, *36*).

### Parental histones are lost upon disabling both leading and lagging strand recycling

**Fig. 2.**
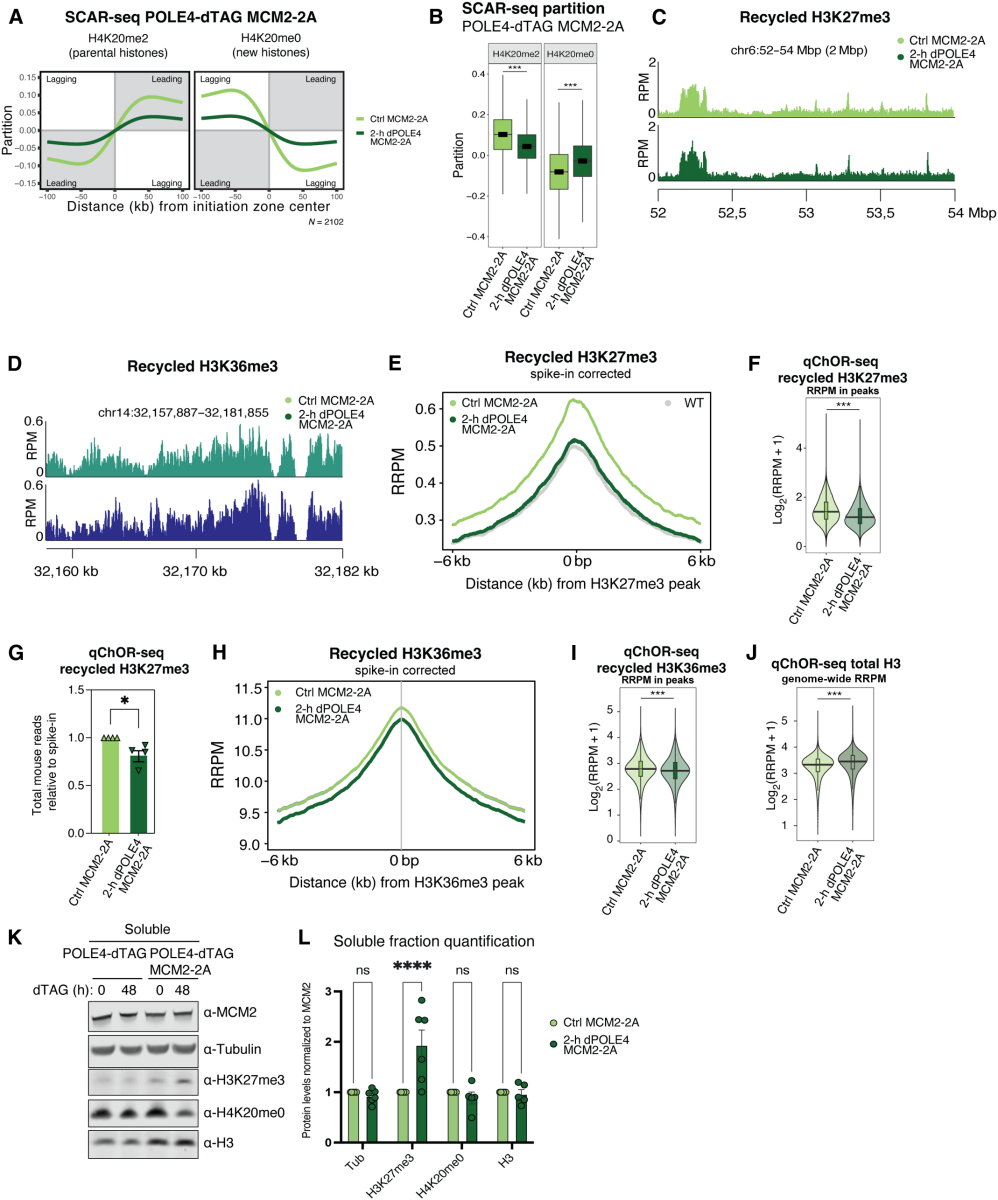
Simultaneous disruption of leading and lagging strand histone recycling results in parental histone loss. Average SCAR-seq profile (**A**) or box plots (**B**) show the H4K20me2 and H4K20me0 partition in 1-kb windows around IZs of DMSO (Ctrl MCM2-2A)–or dTAG-13 (dPOLE4 MCM2-2A)–treated POLE4-dTAG MCM2-2A mESC. *n* = 2 biological replicates. Box plot as in Fig. 1C. Wilcoxon signed-rank test. (**C** and **D**) Occupancy tracks for H3K27me3 (C) or H3K36me3 (D) on nascent DNA in DMSO (Ctrl MCM2-2A, top)–or dTAG-13 (dPOLE4 MCM2-2A, bottom)–treated POLE4-dTAG MCM2-2A mESC. Signal is represented in RPMs. (**E**) Signal of H3K27me3 overlapping the H3K27me3 peaks upon DMSO or 2-hour dTAG-13 treatment in POLE4-dTAG MCM2-2A mESC. *n* = 4 biological replicates. Wild levels (DMSO-treated POLE4-dTAG cells) are shown in gray. (**F**) Average profile of recycled H3K27me3 across total H3K27me3 peaks in DMSO or 2-hour dTAG-13. Signal quantified with reference-adjusted RPMs using exogenous spike-in chromatin (RRPM). *n* = 2 biological replicates. Wilcoxon signed-rank test. (**G**) Bar plot of mapped reads (mouse) relative to spike-in reads (*Drosophila*) for H3K27me3 in POLE4-dTAG MCM2-2A mESC treated with DMSO or 2-hour dTAG-13. *n* = 4 biological replicates. Normalized to EdU input. (**H**) Average profile of recycled H3K36me3 across total H3K36me3 peaks in DMSO or 2-hour dTAG-13. Signal is quantified as in (E). *n* = 3 biological replicates. (**I**) Average profile of recycled H3K36me3 occupancy across H3K36me3 peaks in DMSO (Ctrl MCM2-2A) or dTAG-13 (dPOLE4 MCM2-2A). Analyzed as in (F). *n* = 3 biological replicates. (**J**) Average profile of total H3 occupancy in genome-wide bins after DMSO or dTAG-13 treatment. Analyzed as in (F). *n* = 3 biological replicates. Western blot analysis (**K**) and quantification (**L**) of POLE4-dTAG and POLE4-dTAG MCM2-2A mESC protein soluble fraction treated for 48 hours with DMSO or dTAG-13. Data normalized to MCM2 levels. *n* = 6 biological replicates. *P = 0.00270; ***P < 2.220446e-16; ****P < 0.001.

To interrogate the immediate effects of combined leading and lagging strand recycling defects, we introduced a POLE4-dTAG in MCM2-2A mutant mESCs (fig. S2A). We confirmed the strong bias of parental histone transmission to the leading strand in an MCM2-2A mutant background (Fig. 2, A and B), mirrored by a bias in the deposition of new histones toward the lagging strand. These biases were reduced, but not lost, upon acute depletion of POLE4, as shown by SCAR-seq of parental and new histones marked by H4K20me2 and H4K20me0, respectively (Fig. 2, A and B). As SCAR-seq measures relative occupancy between leading and lagging strands, reduced parental histone asymmetry could indicate either a rescue of parental histone recycling to the lagging strand or a loss of parental histones from the leading strand (fig. S2B). To address this, we used quantitative ChOR-seq as above to measure recycling accuracy and efficiency. Short-term depletion of POLE4 in MCM2-2A cells did not substantially alter recycling accuracy as assessed by the occupancy of parental H3K27me3 (Fig. 2C and fig. S2, C to F) and H3K36me3 occupancy 10 min after replication (Fig. 2D). However, unlike single POLE4 depletion, acute POLE4 loss in MCM2-2A cells reduced the efficiency of recycling for parental H3K27me3 by 20% within cognate H3K27me3 domains (Fig. 2, E and F) and by 15% if considering all reads in nascent chromatin (Fig. 2G). In accordance, the levels of recycled parental H3K36me3 and H4K20me2 were also reduced on nascent chromatin (Fig. 2, H and I, and fig. S2G), while total H3 levels slightly increased (Fig. 2J). This argues that parental histones are lost during replication, potentially with some moderate degree of reincorporation at other sites in the genome as the loss is more moderate when considering the signal of parental histones in total nascent chromatin compared to newly replicated cognate H3K27me3 domains. Consistent with this conclusion, H3K27me3-marked histones could be detected in the soluble pool of free histones when both MCM2 and POLE4 recycling pathways were impaired (Fig. 2, K and L). As total histone levels are not reduced, we anticipated that new histone deposition was compensating for the loss of parental histones. Consistent with this, chromatin accessibility on nascent chromatin was not altered as evaluated by replication transposase-accessible chromatin with sequencing (repli-ATAC-seq) (fig. S2H), which shows strongly elevated accessibility upon acute loss of new histones deposition by chromatin assembly factor 1 (CAF-1) (*37*). Therefore, disabling both leading and lagging strand pathways compromises the efficiency of parental histone transfer to newly synthesized DNA without substantially changing overall nucleosome occupancy on new DNA.

### Loss of histone-based information challenges mESC viability

**Fig. 3.**
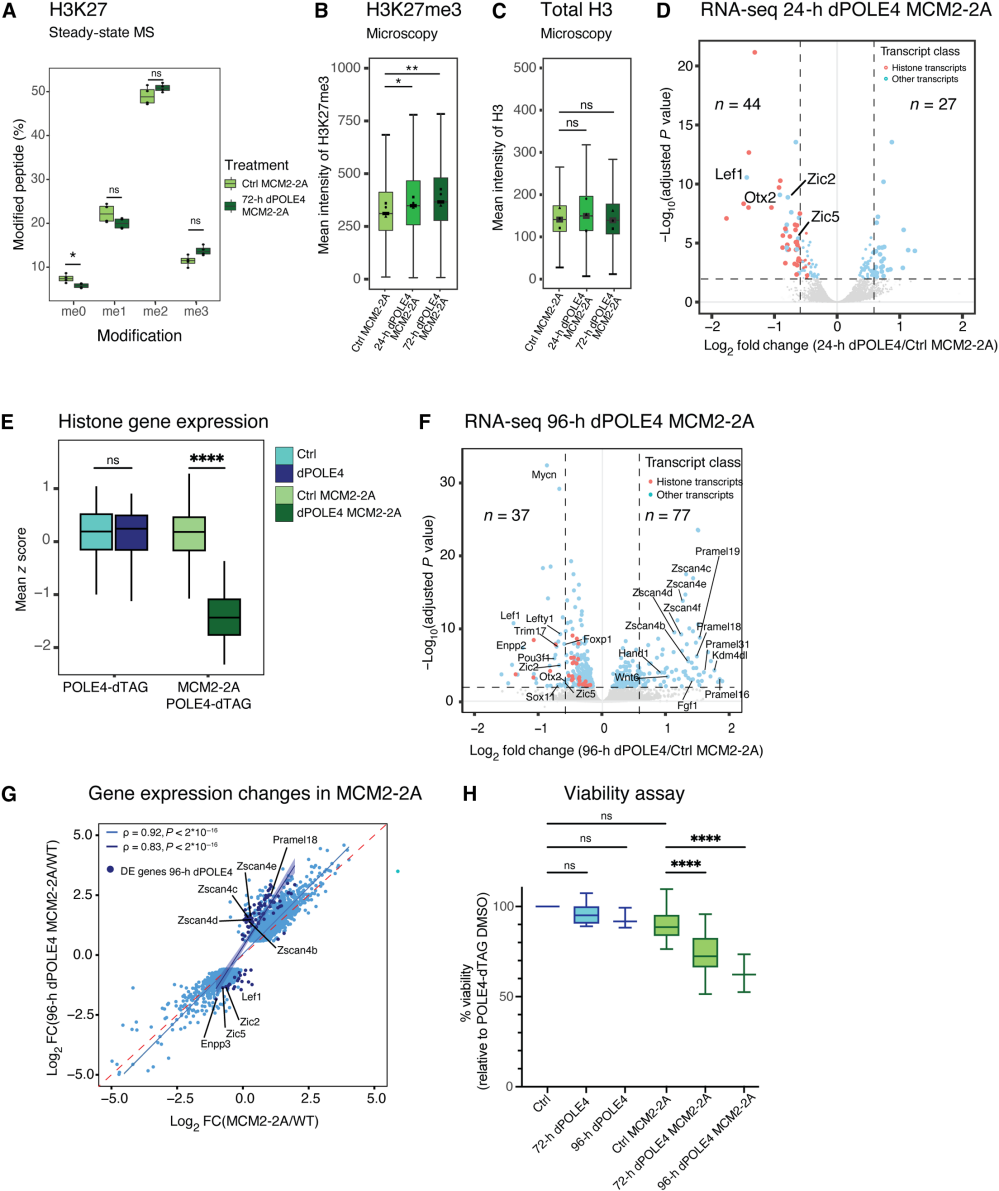
Simultaneous disruption of leading and lagging strand histone recycling results in gain of H3K27me3, decreased expression of histone and formative pluripotency factors, and loss of viability. (**A**) Box plot showing global H3K27 methylation levels quantified by MS. *n* = 4 biological replicates. *P = 0.043 for me0. (**B** and **C**) High-content microscopy of H3K27me3 (B) and H3 (C) levels in POLE4-dTAG MCM2-2A mESC treated with DMSO (Ctrl MCM2-2A) or dTAG-13 (dPOLE4 MCM2-2A). *n* = 3 biological replicates. *P = 0.0423; **P = 0.015. (**D**) Volcano plot showing differential expression analysis of genes and repeat subfamily expression in 24-hour POLE4-depleted MCM2-2A mESC (dPOLE4 MCM2-2A/Ctrl MCM2-2A). Fold change (FC) against false discovery rate (FDR) adjusted *P* value is shown per gene in 24-hour dTAG-13 (dPOLE4 MCM2-2A) versus DMSO (Ctrl MCM2-2A). Significant genes (|log_2_ fold change | > 0.58, adjusted *P* value < 0.01) are depicted in light blue or pink (histone transcripts). *n* = 3 biological replicates. (**E**) Relative RNA level (*z* score normalized) of all histone genes from RNA-seq performed in 2D. Boxplot shows the distribution of histone genes. Wilcoxon signed-rank test. ****P = 8.2e-18. (**F**) Volcano plot showing differential expression analysis of genes and repeat subfamily expression after 96-hour POLE4 depletion in MCM2-2A mESC (dPOLE4 MCM2-2A/Ctrl MCM2-2A). Cutoff and annotation as in (D). (**G**) Scatterplot of DE genes in the MCM2-2A background (MCM2-2A/WT). *Y* axis shows log_2_ fold change of MCM2-2A/WT, and *x* axis shows log_2_ fold change of dPOLE4 MCM2-2A/WT. DE genes after 96-hour POLE4 depletion in MCM2-2A from (F) are highlighted in blue. Spearman correlation coefficient (**ρ**) with *P* value. (**H**) Cell-Titer Blue viability assay of POLE4-dTAG and POLE4-dTAG MCM2-2A mESC with DMSO and dTAG-13 treatment for the indicated times. Data were normalized to DMSO-treated POLE4-dTAG mESC [72 hours, *n* = 4 biological replicates; 96 hours, *n* = 3 biological replicates. Student *t* test (two-tailed, paired)]. ****P < 0.0001.

Next, we compared the phenotypes of the single and double recycling perturbation to understand the consequences of their inheritance defects. With a cis-based asymmetric defect, most pronounced in MCM2-2A mutants, disrupting both pathways (dPOLE4 MCM2-2A) resulted in the loss of parental histones with moderate asymmetry. MS analysis after 72 hours of POLE4 depletion in the MCM2-2A background revealed no changes in the global levels of total histone H3-H4, the replicative histone H3.1 and the replacement variant H3.3, and new and old histones marked by H4K20me0 and H4K20me2, respectively (fig. S3, A to D). However, global levels of H3K36me3 and H3K27me3 increased (Fig. 3A and fig. S3E). High-content microscopy confirmed a progressive increase of H3K27me3, but not H3, upon depletion of POLE4 in the MCM2-2A mutant from about 2 cell cycles and longer (Fig. 3, B and C, and fig. S3F). This was also observed in both MCM2-2A single mutant (*18*, *19*, *36*) and upon POLE4 depletion (Fig. 1F), indicating that this abnormality in chromatin restoration is more generally associated with recycling defects and not strictly linked to parental histone loss. We conclude that the decrease in parental histone recycling on individual strands independently and additively contributes to a global increase in H3K27me3 levels.

To further explore the effects on gene expression, we performed RNA sequencing (RNA-seq). The transcriptional changes in MCM2-2A mutants in comparison to WT recapitulated previous work, including expression of two-cell–like cell (2CLC) genes, deregulation of bivalent genes, and loss of repeat repression (fig. S3G) (*11*, *18*, *19*). While depletion of POLE4 alone for 24 hours did not affect steady-state gene expression (fig. S1C), a number of genes were deregulated in the MCM2-2A background (Fig. 3D). This included the down-regulation of many replication-dependent histone genes, including both core and linker histones, while the expressions of replication-independent histones H3.3 and Cenpa were unchanged (Fig. 3, D and E, and fig. S3H). We ruled out a role of cell cycle changes or DNA damage in these expression changes, as POLE4 depletion did not affect nucleotide analog 5-ethynyl-2′-deoxyuridine (EdU) incorporation, cell cycle distribution, cell division rate, or DNA damage signaling (e.g., yH2AX and pS4/8-RPA) (fig. S3, I to L). Consistently, expression of other cell cycle–regulated genes was unaltered (table S1). However, perturbation of both recycling pathways down-regulated formative pluripotency factors Lef1, Zic2, Zic5, and Otx2 (Fig. 3D), suggesting that the typical transitioning between mESC states (naïve pluripotency, 2CLCs, and lineage priming) is further impaired beyond that reported for MCM2-2A mESCs (*18*). In agreement, we found that a third of the up-regulated genes (11 of 27, for example, *Itgb4*, *Btg2*, and *Abcb1b*) are reported to be suppressed by Zic2 (*38*). Short-term (24 hours) POLE4 depletion did not up-regulate repeat expression further in MCM2-2A mESCs, likely reflecting that MCM2-2A single mutants already show substantial repeat derepression (fig. S3M). Prolonged POLE4 depletion (96 hours) in MCM2-2A background resulted in 118 DE genes, most of which show expression changes in the MCM2-2A alone and become further deregulation upon perturbation of both recycling pathways (Fig. 3, F and G). Most prominently were the 2CLC genes and repeats (e.g., MERVL-int/MT2 and the Zscan4) up-regulation, while formative pluripotency factors remained down-regulated (Fig. 3, F and G). The effect on histone genes is less pronounced upon longer POLE4 depletion (fig. S3N), suggesting that it is an acute response to the loss of parental histones to the soluble pool to which cells can adapt.

The transcriptome analysis indicated that loss of MCM2 and POLE4-mediated recycling has synergistic effects on gene expression in mESCs, linked to impaired transitioning between cellular states. Notably, cellular viability was also compromised upon prolonged POLE4 depletion in the MCM2-2A mutant (Fig. 3H), while disabling either pathway alone did not affect viability.

### Loss of parental histones during DNA replication severely impairs differentiation

**Fig. 4.**
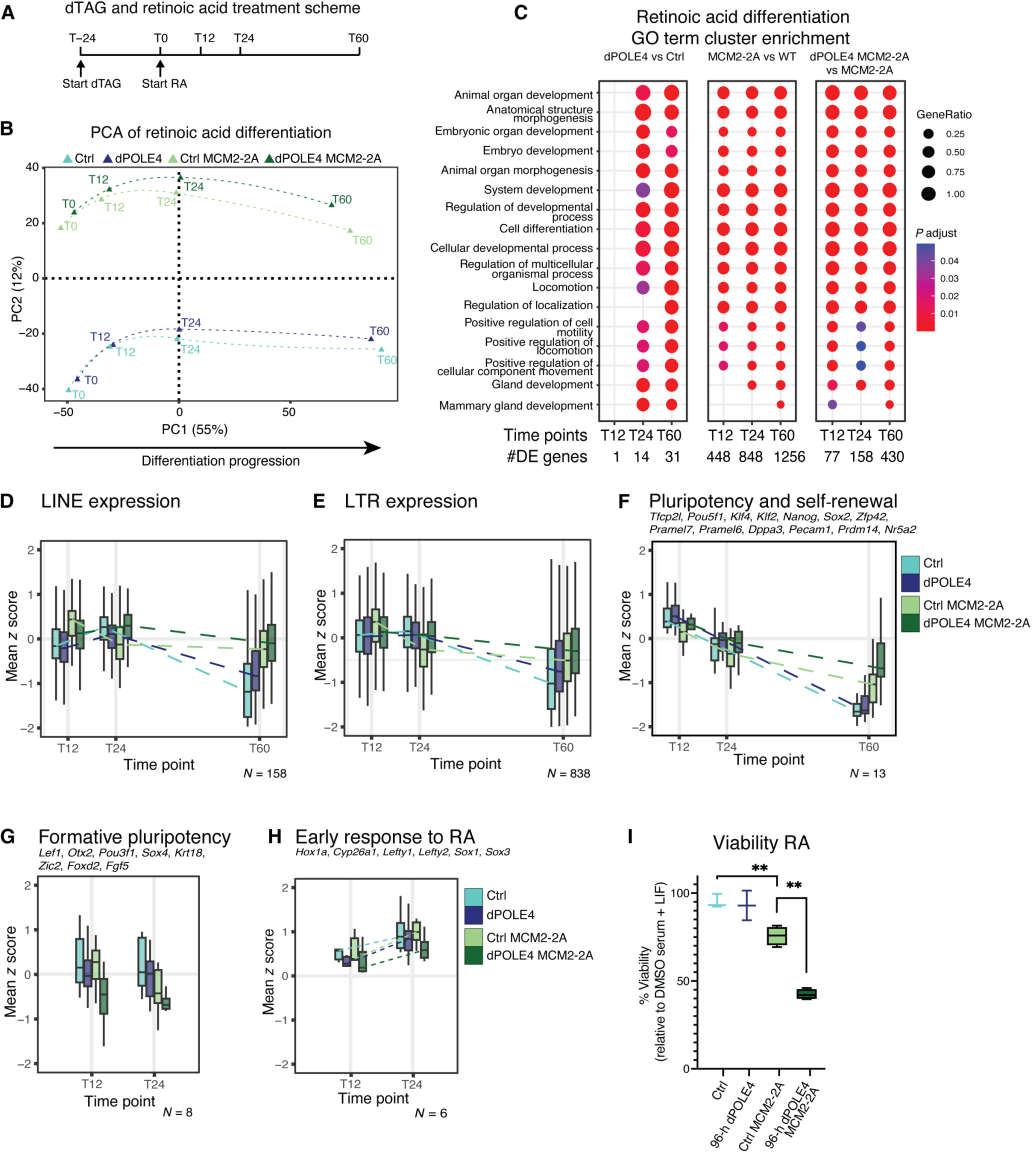
Simultaneous disruption of leading and lagging strand histone recycling during RA differentiation impairs down-regulation of repeat expression and (formative) pluripotency factors. (**A**) Scheme of dTAG and RA treatment and RNA-seq time points. Cells were first grown in fetal bovine serum (FBS) + leukemia inhibitory factor (LIF) conditions and treated with DMSO or dTAG for 24 hours. After 24 hours, cells were grown in medium containing RA without LIF. (**B**) PCA of RNA-seq samples during RA treatment generated from all expressed genes in POLE4-dTAG and POLE4-dTAG MCM2-2A mESC treated with dTAG13 (dPOLE4, dPOLE4 MCM2-2A) or DMSO (Ctrl, Ctrl MCM2-2A). (**C**) Dot plot gene ontology (GO) analysis of biological processes in the down-regulated genes and up-regulated genes in POLE4-depleted mESC (dPOLE4 versus Ctrl), steady-state MCM2-2A mESC (Ctrl MCM2-2A versus Ctrl), and POLE4-depleted MCM2-2A mESC (dPOLE4 MCM2-2A versus Ctrl MCM2-2A). Gene ontology is performed on DE genes uniquely changed upon RA treatment. (**D** to **H**) *Z* score normalized expression analysis of (D) LINE (*n* = 158 genes), (E) LTR elements (*n* = 838 genes), (F) naïve pluripotency and self-renewal genes (*n* = 13 genes), (G) formative pluripotency genes (*n* = 8 genes), and (H) early RA response genes (*n* = 6 genes), in POLE4-dTAG and POLE4-dTAG MCM2-2A mESCs treated with DMSO (Ctrl, Ctrl MCM2-2A) or dTAG13 (dPOLE4, dPOLE4 MCM2-2A). *n* = 3 biological replicates. (**I**) Cell-Titer Blue viability assay of dPOLE4 and dPOLE4 MCM2-2A mESC growing under RA conditions for the indicated treatments. Data normalized to DMSO-treated dPOLE4 mESC in FBS + LIF. Two-way analysis of variance (ANOVA; three biological replicates for dPOLE4 cells and four biological replicates for dPOLE4 MCM2-2A mESC). Ctrl vs Ctrl MCM2-2A (top) **P = 0.0080, Ctrl MCM2-2A vs dPOLE4 MCM2-2A (bottom) **P = 0.0031.

To address differentiation competence in our mutants, we focused on in vitro differentiation toward neural lineage specification in response to all-trans retinoic acid (RA) (*39*, *40*). We depleted POLE4 for 24 hours before RA addition to allow the recycling defect to propagate to the chromatin environment and followed transcription during differentiation (Fig. 4A). Principal components analysis (PCA) of transcriptome data separated differentiation time points in PC1, while PC2 separated MCM2-2A and WT backgrounds (Fig. 4B and fig. S4A). All histone recycling mutants followed similar differentiation trajectories for the first 24 hours but separated at 60 hours, with the dPOLE4 MCM2-2A cells showed the most severe defect (Fig. 4B). The depletion of POLE4 before RA differentiation increased the number of DE genes in both WT and MCM2-2A backgrounds (fig. S4B). Although the number of DE genes varied, they were consistently enriched for differentiation and developmental processes (Fig. 4C). While changes after dPOLE4 in WT background did not correlate to the response to RA (fig. S4C), there was a clear inverse relationship between RA-induced change in WT cells and the histone recycling mutants MCM2-2A and induced POLE4 depletion in MCM2-2A (fig. S4, D and E), indicating that the RA-induced transcriptional program was not properly executed in these recycling mutants. This was also illustrated by focusing on specific transcript types, including DNA repeat expression and the pluripotency network. In WT cells, long terminal repeat (LTR) families and long interspersed nuclear element (LINE) expression decrease during differentiation (*41*). These transcripts are elevated in POLE4-KO and MCM2-2A single mutants in their mESC state (*1*, *18*) and fail to be silenced during differentiation, also in dPOLE4 MCM2-2A cells (Fig. 4, D and E). MCM2-2A mutants failed to shut down the pluripotency network, as evidenced by the expression of key pluripotency factors like *Pou5f1* (Oct4), *Nanog*, *Klf4*, *Tfcp2l1*, or *Esrrb* at the 60-hour time point (Fig. 4F and fig. S4D). This agrees with previous work (*18*, *19*). Notably, this defect was magnified upon POLE4 depletion in MCM2-2A cells (Fig. 4F and fig. S4, E and F). Formative pluripotency factor expression was also perturbed compared to WT cells and mostly so in the MCM2-2A mutants depleted for POLE4, which had low levels of the formative pluripotency factors before differentiation and at early time points T12 and T24 (Figs. 3D and 4G). Moreover, while POLE4-depleted cells and MCM2-2A single mutant up-regulate early differentiation genes like *Sox1*, *Sox3*, *Lefty1*, *Lefty2*, and *Hox1a*, dPOLE4 MCM2-2A cells lack a robust early differentiation response (Fig. 4H). This is consistent with the view that dPOLE4 MCM2-2A cells are less able to switch between cellular states required for differentiation (*40*, *42*, *43*). Underscoring this, the loss of POLE4 in MCM2-2A cells severely challenged cellular viability during RA-induced differentiation beyond what was observed in self-renewing conditions (Fig. 4I). In summary, simultaneous loss of POLE4 and MCM2 recycling pathways further impairs both repeat silencing and the robust and timely transitions in expression programs toward differentiation, including the shutdown of pluripotency and activation of differentiation genes. This manifested in a marked reduction of cell viability, illustrating synthetic lethality upon disabling both lagging and leading strand recycling in cells undergoing differentiation.

## DISCUSSION

In this study, we demonstrate the synergistic effect of impairing parental histone transmission to both leading and lagging strands. Our inducible models provided us the opportunity to elucidate the sequence of events following the acute perturbation of histone recycling to the leading strand in a WT or MCM2-2A mutant background. Disruption of both POLE4 and MCM2-mediated recycling pathways leads to a substantial loss of parental histones during replication due to their release into the soluble fraction. This reduced transmission of histone-based information exacerbates gene expression and differentiation defects in MCM2-2A mutants and synthetically challenges cell viability. We further reveal that the accumulation of H3K27me3, a hallmark of histone recycling mutants (*18*, *19*, *36*, *44*), is an early response to impaired recycling (in single and double mutants) preceding gene expression changes. Together, our findings support that the overall abundance of parental histones postreplication is important for epigenome restoration, underscoring the pivotal role of parental histone recycling in genome regulation and supporting transcriptional programs driving cellular specification.

We find that parental histones are lost during replication in dPOLE4 MCM2-2A cells, indicating a loss of epigenetic information consistent with work in budding yeast (*45*–*47*). In contrast, combined abrogation of POLE3/4 and MCM2-dependent histone recycling in fission yeast rescued the silencing defects of the individual mutants (*48*), suggesting compensation through a mechanism that might be specific to the fission yeast replisome. Upon disabling either POLE4- or MCM2-mediated recycling in mESCs, parental histones are not lost but rerouted toward either the lagging or leading strand [this work and (*18*)], and as such, the overall amounts of parental histones in replicated domains remain unchanged. Some modifications show enhanced recycling in the single POLE4 depletion mutant, consistent with previous observations using orthogonal methods (*9*). This suggests that not all parental histones are recycled with the same efficiency in WT cells, which could be dependent on factors such as replication fork speed or chaperone activity, also supported by observations that recycling efficiency is enhanced in the absence of de novo histone deposition (*49*). The lack of parental histones in cis on either strand alters chromatin restoration and deregulates the epigenome, resulting in gene expression changes and differentiation defects (*18*, *19*), scaling with the degree of asymmetry (this work). When POLE4 is depleted in MCM2-2A cells, about 20% of the parental histones are lost during replication. According to the SCAR-seq analysis, most of the remaining parental histones (about 80%) are being transmitted to the leading strand compared to 50% in WT cells. We therefore anticipate that the leading strand may not experience lower levels of parental histones than in WT cells with symmetric recycling, while the lagging strand likely is impaired comparably to MCM2-2A mutation alone. The exacerbation of the phenotype upon POLE4 depletion in MCM2-2A cells thus hints toward nonstrand-specific effects of histone recycling, linked to the overall concentration of parental histones in postreplicative chromatin. This is consistent with ideas that histone modifications can propagate through mechanisms linked to the three-dimensional (3D) organization of chromatin (*31*), such as segregation of biochemical activities in compartments or condensates (*50*, *51*) and read-write activity in 3D (*30*, *52*), potentially including cross-talk between sister chromatids.

The degree of histone recycling asymmetry is substantially more pronounced in MCM2-2A mutants compared to mESCs lacking POLE4 (*1*, *3*, *11*, *18*), indicating the existence of additional routes for transmission of histones to the leading strand. Consistent with this, the dPOLE4 MCM2-2A cells show a moderate leading strand recycling bias along with loss of parental histone to the soluble pool as discussed above. Previous work by enhanced S-phase progression analysis by nascent DNA sequencing (eSPAN) did not detect such moderate level of asymmetry in the double MCM2-2A and POLE4-KO mutants (*35*, *48*), likely reflecting higher sensitivity of SCAR-seq that also requires more material. We conclude that acute failure in leading strand recycling due to depletion of POLE4 results in a partial loss of parental histones otherwise rerouted to the leading strand in MCM2-2A mutants. Yet, a substantial proportion of parental histones continues to be recycled to the leading strand, and we predict that full abrogation of this pathway would further exacerbate the phenotype including cell viability. Mrc1/CLASPIN was recently discovered as a regulator of histone recycling to both leading and lagging strands (*14*–*16*), and we identified a CLASPIN deletion mutant in mESCs with defective leading strand recycling (*15*). Given the strong asymmetry in this mutant, comparable to MCM2-2A mutants but of opposite directionality, CLASPIN mutations provide a new tool to manipulate leading strand recycling that will complement POLE4/3 deletion.

Recent reports demonstrate that H3K9me3-mediated repeat silencing is highly dependent on the transmission of parental histones during DNA replication (*11*, *18*, *53*). In line with this, the inability to shut down repeat expression during differentiation in the single and double recycling mutants argues that the establishment of H3K9me3 silencing across multiple cell generations requires the inheritance of parental histones. This is particularly prominent for LTR repeats that can harbor binding sites for pluripotency factors, and for which derepression therefore has consequences for the gene regulatory network governing pluripotency (*54*, *55*). Accordingly, acute short-term (24 hours) POLE4 depletion in MCM2-2A cells challenges the lineage-primed state by the down-regulation of several formative pluripotency factors, and longer depletion reveals further misregulation of 2CLC genes, known to be regulated by H3K9 methylation, and developmental genes bivalent for H3K27me3 and H3K4me3. This further destabilization of the gene expression pattern typical to self-renewing embryonic stem cells could underpin the loss of viability in these cells. Additional work is needed to explore this relationship, as there is no evidence of cell cycle arrest and DNA damage. dPOLE4 MCM2-2A cells also fail to differentiate toward the neuronal lineage in response to RA, and cell viability is further challenged in this process. Loss of histone-based information affects repeat repression, self-renewal, and the robust transition from pluripotency toward lineage specification. This corroborates recent findings that faithful histone inheritance is pivotal for early embryonic development (*19*, *56*). Whether differentiated cells, relying more on DNA methylation, will have the same dependency on histone-based inheritance for genome regulation will be important to address.

Apart from developmental genes, short-term depletion (24 hours) of POLE4 in MCM2-2A cells caused a decrease in replicative histone transcripts. This occurs concomitantly with the release of some parental histones into the soluble, nonnucleosomal histone pool. This is unlikely to reflect specific epigenetic deregulation at the replicative histone genes due to impaired histone recycling. The soluble pool of histones is under tight regulation by histone chaperones to match histone supply with demand via feedback control of histone mRNA transcription, processing, and/or stability (*37*, *57*–*59*). We thus anticipate that a release of parental histones could lead to the down-regulation of histone mRNAs. This would parallel the response to CAF-1 depletion where soluble histones cannot be incorporated into chromatin, and replicative histone transcripts are down-regulated (*37*). Consistent with this view, down-regulation of histone transcripts appears to be an acute response, as it is less pronounced at later time points. Total histone levels measured by MS do not change, even after prolonged POLE4 depletion in an MCM2-2A mutant background, arguing that a new equilibrium is established, potentially through compensation at the posttranscriptional level. This is in agreement with the notion that new histone deposition compensates for the loss of parental histones, supported by SCAR-seq where new histone occupancy inversely correlates with old histones. It is also possible that the evicted parental histones enter the supply chain for new histones via Nuclear Autoantigenic Sperm Protein (NASP) or Anti-Silencing Function 1 (ASF1) and become incorporated at other replication sites through CAF-1–mediated de novo deposition (H3.1/2-H4) or via Histone Regulator A (HIRA)-dependent gap filling (H3.3-H4) (*60*). In asynchronous cell populations, we could not detect changes in the relative proportion of new and old histones or replicative histone H3.1 compared to the replacement variant H3.3. This would be consistent with the idea that old, evicted histones would reenter the supply chain. Whether this involves parental histones being stripped of their modifications before reincorporation remains to be explored.

Somewhat counterintuitively, cells show aberrant accumulation of H3K27me3 in response to POLE4 depletion in both WT and MCM2-2A cells and thus regardless that recycling efficiency is hampered in the latter setting. This accumulation is not observed in nascent chromatin (where H3K27me3 is lost in dPOLE4 MCM2-2A) but reflects aberrant H3K27me3 modification of new histones during chromatin restoration consequent to the reduced contribution of parental histones on one or both strands. Elevated levels of H3K27me3 were previously reported in both MCM2-2A and POLE4-KO mESCs and in MCM2-2A MCF7 cells (*18*, *19*, *36*, *44*) and proposed to be upstream of gene expression changes in mESCs (*18*) and upon differentiation (*19*). The H3K27me3 increase in single mutants reflects altered levels at promoters of bivalent genes (*18*, *19*) as well as low levels of epigenetic noise outside of cognate polycomb domains (*18*). The use of an inducible system now reveals that the global increase in H3K27me3 is a relatively fast response to histone recycling defects, occurring within the first 1 to 2 cell cycles after POLE4 depletion. This response precedes gene expression changes in POLE4-depleted cells, univocally linking H3K27me3 deregulation to defective histone recycling. We previously proposed that H3K27me3 accumulation in MCM2-2A cells reflects the lack of negative and positive feedback from parental histones required on both daughter strands to focus and fine-tune the activity of PRC2. The PRC2 subunit SUZ12 showed increased binding to the sister chromatid mainly composed of new histones in MCM2-2A cells (*18*), and H3.3 was found to have a similar bias due to elevated de novo deposition on the strand devoid of parental histones (*19*). Our finding that depletion of POLE4 in an MCM2-2A background further increases H3K27me3 levels supports that this response is a general consequence of impaired inheritance, driven by the lack of parental H3-H4 transfer. PRC2 recruitment is complex and involves combined DNA and nucleosome binding with a preference for GC-rich sequences (*61*) as well as a positive contribution of H3.3 and its chaperone HIRA (*62*). A role for DNA accessibility and H3.3 levels is supported by a recent report that H3K27me3 levels increase upon acute depletion of CAF-1 and deposition of H3.1 which is partly compensated by H3.3 deposition (*37*). Together, our data and the existing literature are consistent with a model where lack of negative feedback from parental histones and elevated opportunity for DNA binding along with increased H3.3 deposition promote aberrant accumulation of H3K27me3.

## MATERIALS AND METHODS

### Cell culture, viability, and differentiation assays

#### Cell culture

WT, MCM2-2A, POLE4-dTAG, and MCM2-2A/POLE4-dTAG mouse ESCs used in this study were derived from the E14JU cell line with a 129/Ola background. For genome editing and next-generation sequencing experiments, mESCs were grown on gelatin-coated dishes (0.2%) in serum + leukemia inhibitory factor (LIF) conditions at 37°C with 5% CO_2_. Media was prepared by supplying DMEM19 GlutaMAX pyruvate with fetal bovine serum (FBS; 15%), LIF (made in-house), 1× nonessential 20 amino acids (Gibco), 1× penicillin/streptomycin (Gibco), and β-mercaptoethanol (0.1 μM). Cells were passaged using trypsin-EDTA (Gibco) or TrypLE (Gibco). Cells were routinely tested for mycoplasma contamination. *Drosophila* S2-DRSC cells were obtained from the Drosophila Genomics Research Center. S2 cells were grown in suspension in spinners in M3 + bactopeptone and yeast extract (BPYE) media: Shields and Sang M3 Insect Medium (Sigma-Aldrich, S-8398), KHCO_3_ (Sigma-Aldrich, 12602), yeast extract (Sigma-Aldrich, Y-1000), bactopeptone (BD, 211705), 10% heat-inactivated fetal calf serum (GE Hyclone, SV30160.03), and 1× penicillin/streptomycin (Gibco, 151400122). Cells were incubated at 25°C with 5% CO_2_.

#### Viability assays

Around 2500 cells were plated on a gelatinized 96-well plate in 100 μl of FBS + LIF media. Four hours before end-point measurements, 20 μl of CellTiter Blue (Promega) was added and incubated at 37°C, 5% CO_2_. Fluorescence intensity was measured using a 560-nm excitation and 590-nm emission filter on a BMG LABTECH CLARIOstar plate reader. For direct comparison, POLE4-dTAG and POLE4-dTAG MCM2-2A cell lines were assayed together.

#### Differentiation assays

RA differentiation of mESCs was performed by plating 3 × 10^5^ cells on a gelatinized 24-well plate in 2 ml of FBS + LIF media. After 24 hours, the medium was replaced by medium without LIF supplied by 1 μM RA. 

### Genome editing

POLE4-dTAG cells were generated in WT and MCM2-2A by CRISPR-Cas9 using the SpCas9(BB)-2A-Puro (PX459) V2.0 plasmid (Addgene #62988) as described in (*63*) with single guide RNA (sgRNA) #1 (ctttggattAATTGTGGAGC), targeting the Pole4 gene at the end of the ORF and a Pole4-linker-dTAG homology donor plasmid (cloned from a BRCA1-Associated Protein 1 (BAP-1) homology donor plasmid, a gift from the Klose lab). Cells were transfected using Lipofectamine 3000 with 0.5 μg of the sgRNA plasmid and 2 μg of the donor plasmid. Cells were sparsely seeded on a 10-cm dish 24 hours posttransfection and selected with puromycin (2 μg/ml) for 48 hours. After 1 week of culture, individual clones were picked manually with a pipette, and each clone was distributed between two 96-well plates (one plate for genotyping and one plate for expansion). For genotyping, cells were washed in phosphate-buffered saline (PBS) and placed at −80°C for more than 30 min. Subsequently, cells were scraped off with 50 μl of squishing buffer [10mM tris (pH 8), 1 mM EDTA, 25 mM NaCl, and proteinase K (200 μg/ml)], transferred to polymerase chain reaction (PCR) tubes, and incubated for 1 hour at 65°C followed by 10 min at 95°C. The resulting genomic DNA (gDNA) was genotyped using One Taq Hot Start 2x Master Mix (New England Biolabs) and indicated primers (forward: AGGAGCCAAGTTCAGTTCCA; reverse: CCCAAACAGCATCAGCTACA). The product was Sanger-sequenced to confirm the integration of the dTAG.

### Cell fractionation

Soluble and chromatin extracts were generally prepared as in (*64*). Briefly, cell pellets were washed twice in PBS, then extracted in ice-cold chromatin wash buffer (ChWB) [ChWB: NaCl (300 mM), NP-40 (0.5%), Hepes·NaOH or tris·HCl (50 mM, pH 7.9/7.6), EDTA (0.2 mM), glycerol (5%), Sodium Fluoride (NaF) (5 mM), β-glycerolphosphate (10 mM), phenylmethanesulfonyl fluoride (0.1 mM), leupeptin (10 μg/ml), pepstatin A (10 μg/ml), trichostatin A (100 ng/ml), and Na_3_VO_4_ (0.2 mM)], transferred to fresh tubes after centrifugation (2800*g*, 3 min, 4°C), and filtered (0.45 mm, using Millipore Ultra-Free MC spin filters). Chromatin pellets were washed with ChWB, spun down (2800*g*, 3 min, 4°C), and digested with Benzonase (0.015 volumes, 25 U/ml, Millipore, 70746, 1 hour, 37°C) in 1 volume of ChWB supplemented with MgCl_2_ (0.01 volume, 1 M). Resultant chromatin extracts were spun down (16,000*g*, 3 min, 4°C), and supernatants were transferred to fresh tubes. Protein concentrations, measured using the Pierce 660nm Protein Assay Reagent (Thermo Fisher Scientific), were equalized before Western blot analysis.

### Western blotting

Whole cell extracts were made by washing cells once with PBS, and Laemmli Sample Buffer was added directly to the well. Lysed cells were scraped off the well into a tube, 25U Benzonase (Sigma-Aldrich) was added, and the lysate was incubated for 1 hour at 37°C before being boiled for 10 min at 95°C. SDS–polyacrylamide gel electrophoresis (SDS-PAGE) and Western blotting were subsequently performed as described in (*3*). Primary antibodies were used in Tris-Buffered Saline with Tween-20 (TBST )and 5% milk at the following concentrations: rabbit anti-POLE4 [1:5000; (*9*)], rabbit anti-H3K27me3 (1:1000; Cell Signaling, #9733S), rabbit anti-MCM2 (1:5000; Cell Signaling, #3619), rabbit anti-H4K20me0 (1:1000; Abcam, ab227804), mouse anti-H4K20me2 (1:1000; Diagenode, C15200205), rabbit anti-PCNA (1:1000; Abcam, ab29), rabbit anti-yH2AX (1:5000; Millipore, JBW301), rat anti-tubulin (1:5000; Abcam, ab6160), mouse anti-H3 (1:5000; Abcam, ab10799), rabbit anti-pRPA32 (1:1000; Bethyl, A300-245A) and incubated with the membrane on a roller overnight at 4°C. The next day, membranes were washed three times with PBST (PBS with 0.3% Triton X-100) and incubated with a secondary antibody for 60 min TBST and 5% milk at room temperature using LI-COR Odyssey IRDye 680RD (1:10,000) or LI-COR Odyssey IRDye 800CW (1:10,000). Membranes were subsequently scanned on a LI-COR Odyssey IR Imager (Biosciences). Western blot was quantified using ImageJ.

### MS of histone PTMs

Cells were seeded in 15-cm dishes (5 × 10^6^ cells per dish, one dish per cell line) and collected 2 days later by trypsinization and washing in PBS. Cell pellets (3 × 10^6^ cells) were snap-frozen and shipped on dry ice to EpiQMAx GmbH. Sample preparation and MS analysis were performed according to the EpiQMAx GmbH protocols. Briefly, acid-extracted histones were resuspended in Lämmli buffer and separated by a 14 to 20% gradient SDS-PAGE and stained with Coomassie (Brilliant blue G-250, 35081.01). Protein bands in the molecular weight range of histones (1523 kDa) were excised as single band/fraction. Gel slices were destained in 50% acetonitrile/50 mM ammonium bicarbonate. Lysine residues were chemically modified by propionylation for 30 min at room temperature with 2.5% propionic anhydride (Sigma-Aldrich, 8.00608) in ammonium bicarbonate (pH 7.5). Subsequently, proteins were digested with 200 ng of trypsin (Promega, V5111) in 50 mM ammonium bicarbonate overnight, and the supernatant was desalted by C18 Stagetips (reversed-phase resin) and carbon Top-Tips (Glygen, TT1CAR) according to the manufacturer’s instructions. After desalting, the eluent was speed-vacuumed until dryness and stored at −20°C until MS analysis.

#### Liquid chromatography–mass spectrometry analysis of histone modifications

Peptides were resuspended in 17 μl of 0.1% trifluoroacetic acid. A total of 5.0 μl was injected into a nano–high-performance liquid chromatography device (Thermo Fisher Scientific, Ultimate Nano3000) using a gradient from 4% B to 90% B (solvent A 0.1% Formic Acid (FA) in water, solvent B 80% Acetonitrile (ACN), 0.1% FA in water) over 90 min at a flow rate of 300 nl/min in a C18 Ultra-High Pressure Liquid chromatography (UHPLC) column (Thermo Fisher Scientific, 164534). Data were acquired in parallel-reaction monitoring (PRM)-positive mode using a Q Exactive HF spectrometer (Thermo Fisher Scientific) to identify and quantify specific N-terminal peptides of histone H3 and histone H4 proteins and their PTMs. One survey MS1 scan and nine MS2 acquisitions precursor mass/charge ratio (*m/z*) value in the inclusion list was performed. MS1 spectra were acquired in the *m/z* range of 250 to 1600 with resolution 30,000 at *m/z* 400 [Automatic Gain Control (AGC) target of 3 × 10^6^]. PRM spectra were acquired with a resolution of 15,000 to a target value of 2 × 10^5^, a maximum injection time (IT) of 60 ms, and an isolation 2 window of 0.7 *m/z* and fragmented at 27% normalized collision energy. Typical mass spectrometric conditions were as follows: spray voltage, 1.5kV; no sheath and auxiliary gas flow; heated capillary temperature, 250°C.

#### MS data analysis and quantification of histone modifications

Raw files were searched with the Skyline software against histone H3 and H4 peptides and their respective PTMs with a precursor mass tolerance of 5 parts per million. The chromatogram boundaries of +2 and +3 charged peaks were validated, and the total area MS1 under the first four isotopomers was extracted and used for relative quantification and comparison between experimental groups. The total area MS1 of coeluting isobaric peptides (i.e., H3K36me3 and H3K27me2K36me1) was resolved using their unique MS2 fragment ions. The averaged ratio of analogous ions (i.e., y7 versus y7) was used to calculate the respective contribution of the precursors to the isobaric MS1 peak.

Relative abundances (percentages) were calculated as in the following example for H3K18 acetylation: %H3K18ac = (H3K18ac_K23un + H3K18ac_K23ac)/(H3K18un_K23un + H3K18ac_K23unmod + H3K18un_K23ac + H3K18ac_K23ac), where “ac” indicates acetylation and “un” indicates unmodified.

### Immunofluorescence

mESCs were treated with dimethyl sulfoxide (DMSO) or dTAG for 24 or 72 hours, grown on laminin-coated plates (LN511, BioLamina) for the last 16 hours before pulsed in EdU-containing media (10 μM) for 10 min, washed with cold PBS, immediately fixed for 15 min in 4% paraformaldehyde at room temperature, and stored in PBST. Cells were permeabilized by treating with 0.5% Triton in PBS for 20 min. After washing twice with 3% bovine serum albumin (BSA) in PBS, Click-it was performed using the Click-iT Plus Alexa Fluor 647 Picolyl Azide Toolkit (Thermo Fisher Scientific) according to manufacturer’s protocol. After three washes, samples were blocked with blocking buffer (0.1% Triton, 3% BSA, and 10% FBS in PBS) for 1 hour. Next, samples were stained with H3K27me3 antibody (Cell Signaling, #9733S) and H3 antibody (Abcam, ab10799) in blocking buffer overnight at 4°C. Next day, samples were washed with blocking buffer and incubated with secondary antibodies for 1 hour. After washing three times in blocking buffer, samples were stained with 4′,6-diamidino-2-phenylindole (DAPI; 1:10,000) in PBST. Images were acquired with a ScanR high-content screening microscope (Olympus). Automated and unbiased image analysis was carried out with the ScanR analysis software (version 2.8.1). Individual cells were identified on the basis of DAPI staining, and mean and total pixel intensity was measured for each channel. Data were exported and processed using Spotfire software (version 12.1.1; Tibco). At least 5000 cells were quantified per biological replicate for each condition. Visualization of results was done using using R (v4.2.2) in RStudio (v2023.12.1.402). Cell cycle gates were defined using mean EdU and total DAPI intensities.

### CellTrace Violet proliferation assays

Cell labeling with CellTrace Violet was performed according to the protocol provided by the manufacturer (CellTrace Violet Cell Proliferation Kit, Invitrogen). A 5 mM stock solution was freshly prepared, and 2.4 million cells were resuspended in PBS containing 5 μM stock solution. Cells were incubated at 37°C and protected from light for 20 min. Labeling was stopped by adding 5 volumes of culture medium and incubating at 37°C for 5 min. Cells were spun and resuspended again in culture medium before plating them onto six-well plates. To analyze samples, cells were harvested after the indicated culture timest and fixed in 4% formaldehyde for 20 min. Flow cytometry data were acquired using a 5 laser BD FACS Symphony S6 (355, 405, 488, 561, and 640 nm) running FACSDiva 9.5.1 from BD Biosciences, and data analysis was performed in FlowJo (v10.7.1).

### Sister chromatids after replication by sequencing

A step-by-step protocol is available (*34*). Briefly, nascent SCAR-seq samples were prepared from POLE4-dTAG and MCM2-2A/POLE4-dTAG cells in two biological replicates for each histone PTM. Cells were treated with DMSO or dTAG-13 for 2 hours, pulsed in EdU-containing media (10 μM) for 10 min, and harvested immediately. For sample collection, media was aspirated, plates were washed two times with room temperature (RT) PBS, and ice-cold PBS was added to the dishes. Cells were scraped in a cold room and collected by centrifugation, followed by nuclei isolation. Nuclei were aliquoted, snap-frozen, and stored at −80°C until further use. For Micrococcal Nuclease (MNase) digest, nuclei were counted manually using Kova Glasstic Slides, and 2U MNase (Worthington) was added per 1 × 10^6^ nuclei. Digests were performed at 30°C for 20 min. For native chromatin immunoprecipitation (ChIP), 30 to 50 μg of chromatin was used per sample and incubated with antibodies in a total volume of 600 μl overnight at 4°C with a H4K20me2 antibody (Diagenode, C15200205) or a H4K20me0 antibody (Abcam, ab227804). Magnetic beads [anti-rabbit immunoglobulin G (IgG) or anti-mouse IgG Dynabeads, Invitrogen] were added the next morning, and samples were incubated for 2 hours. After three washes each with ice-cold radioimmunoprecipitation assay (RIPA) buffer and low-salt RIPA 0.5 M NaCl buffer, DNA was eluted and purified using the MinElute Reaction Cleanup kit (QIAGEN). Mononucleosomal-sized fragments were isolated by double-sided size selection with AMPure XP beads (Beckman Coulter). EdU-labeled DNA fragments were biotinylated using Click-iT chemistry using Biotin-TEG-Azide (Berry & Associates). Libraries were prepared using the KAPA Hyper Prep Kit (Roche). Biotinylated fragments were captured using Dynabeads MyOne Streptavidin (Invitrogen), and EdU strands were purified by performing NaOH washes. Libraries were amplified from EdU strands in 9 to 11 PCR cycles. Libraries with mononucleosomal-sized inserts were isolated by double-sided size selection with AMPure XP beads (Beckman Coulter), followed by a second clean-up with 1.0× AMPure XP beads. Fragment distribution of libraries was checked on a Fragment Analyzer system (Agilent). Stranded input samples were prepared in parallel with SCAR-seq samples. Samples were sequenced single-end (75 bp) on a NextSeq500 instrument (Illumina). For direct comparison, dPOLE4 and dPOLE4 MCM2-2A cell lines were assayed together.

#### Data processing

Reads were processed and mapped, and histone partition signal was computed as described previously (*3*). Briefly, for each strand, the SCAR normalized signal in counts per million (CPM) was computed in 1-kb bins and smoothed in a uniform blur considering the neighboring 30 bins on each side. For each 1-kb window, the signal from its corresponding SCAR input was subtracted, and negative values were set to zero. Input-corrected windows with CPM < 0.3 on both strands were filtered out and not considered for further analyses. The final partition score for each 1-kb window was calculated as: Partition = (*F* − *R*)/(*F* + *R*) where *F* and *R* correspond to the number of normalized and input-corrected reads for the forward and reverse strands, respectively. The partition value relates to the ratio of histones with a specific modification being segregated to the nascent forward (partition > 0) or nascent reverse (partition < 0) strand within each window, respectively. Okazaki-seq replication fork directionality scores and filtered initiation zones for mESCs were taken from (*3*) and used to define replication via leading or lagging strand mechanism. In the case xGen UDI-UMI adapters are used for SCAR library preparation, please note that the complementary strand will be sequenced (https://eu.idtdna.com/pages/support/faqs/can-the-xgen-unique-dual-index-umi-adapters-be-used-for-rna-seq). This results in a seemingly mirrored partition ratio (*R* − *F*)/(*R* + *F*) that should be corrected by multiplication with −1.

### Chromatin occupancy after replication

A step-by-step protocol is available (*34*). Briefly, nascent ChOR-seq samples were prepared from POLE4-dTAG and MCM2-2A/POLE4-dTAG cells in three biological replicates. Cells were treated with DMSO or dTAG-13 for 2 hours, pulsed in EdU-containing media (10 μM) for 20 min, and fixed in 7 ml of fixation buffer A (truChIP Chromatin Shearing Kit, Covaris, 520127), and 11.1% formaldehyde was added to a final concentration of 1% for 5 min with constant movement at room temperature. The reaction was quenched by adding glycine to a final concentration of 0.1 M for 5 min. Fixed cells were washed twice in ice-cold PBS and then collected in ice-cold PBS using a cell lifter before centrifuging for 5 min at 500*g* at 4°C. Cells were transferred to Eppendorf tubes, centrifuged (5 min at 500*g* at 4°C), snap-frozen in liquid nitrogen, and stored at −80°C until lysis. Nuclei isolation was performed on fixed cells using the truChIP Chromatin Shearing Kit (Covaris, 520127) following the manufacturer’s instructions. Twenty million cells were sonicated in 1-ml tubes using a Covaris S220 with the following settings: duty cycle, 10% intensity; 200 cycles per burst; 20-min processing time; 7°C bath temperature; and water level full. Sonicated chromatin was centrifuged at 14,000 rpm at 4°C for 10 min, and the supernatant was used for subsequent steps. In parallel, *Drosophila* S2 cells were labeled with 10 μM EdU for 39 hours, fixed, lysed, and sonicated. After sonication, input chromatin was mixed with EdU-labeled *Drosophila* S2 chromatin (0.05% of total chromatin). For each ChIP, 30 μg for H3K27me3 and 45 μg for H3K36me3, H4K20me2 and H3 of mixed mESC and *Drosophila* S2 sonicated chromatin were diluted up to 500 μl with dialysis buffer [4% glycerol, 10 mM tris-HCl, 1 mM EDTA, and 0.5 mM EGTA (pH 8)] and 400 μl of incubation buffer [2.5% Triton X-100, 0.25% sodium deoxycholate, 0.25% SDS, 0.35 M NaCl, and 10 mM tris-HCl (pH 8)] supplemented with leupeptin, aprotinin, pepstatin, trichostatin A, and phenylmethylsulfonyl fluoride (PMSF). For H3K27me3, chromatin was precleared with Protein A Agarose beads (Thermo Fisher Scientific) for 1 hour at 4°C. Next, 10 μl was set aside as input, and the remaining chromatin was incubated with 30 μl of H3K27me3 antibody (Cell Signaling, #9733S), H3K36me3 (Abcam, ab9050), H4K20me2 (Diagenode, C15200205), or H3 (Abcam, ab1791) overnight at 4°C. The following day, the mix was incubated for 3 hour with preblocked Protein A Agarose beads [incubated in BSA (1 mg/ml) in RIPA buffer overnight] for H3K27me3 and with Dynabeads Protein A (Thermo Fisher Scientific, 1001D) for H3K36me3, H4K20me2 and H3. Subsequently, ChIPs were washed three times in ice-cold RIPA buffer [140 mM NaCl, 10 mM tris-HCl, 1 mM EDTA, 1% Triton X-100, 0.1% SDS, 0.1% sodium deoxycholate, and 1 mM PMSF (pH 8)], three times in RIPA buffer with 0.5 M NaCl, once in LiCl buffer [250 mM LiCl, 10 mM tris-HCl, 1 mM EDTA, 0.5% IGEPAL CA-630, and 0.5% sodium deoxycholate (pH 8)], and twice in TE [10 mM tris-HCl and 1 mM EDTA (pH 8)]. The set-aside inputs and the washed beads were incubated with 50 μg of ribonuclease A (RNase A; Sigma-Aldrich) for 30 min at 37°C; thereafter, SDS and NaCl were added to a final concentration of 0.5% and 100 mM, respectively, and samples were incubated with proteinase K (10 μg) for 10 hours at 37°C followed by 6 hours of incubation at 65°C for decross-linking. DNA was purified using the MinElute PCR purification kit (QIAGEN, 28004). All immunoprecipitated DNA or 20 ng of decross-linked input material was subjected to end repair, A-tailing, and adaptor ligation using the KAPA Hyperprep kit following the manufacturer’s instructions and cleaned up with Agencourt AMPure XP beads. For qChOR-seq experiments, indexed DNA from all IP and all inputs were then pooled. Then, Click-iT was performed on indexed and mixed DNA for 30 min at room temperature under the following conditions: 1× Click-iT buffer (Click-iT EdU Alexa Fluor 488 Imaging Kit, Thermo Fisher Scientific, C10337), 0.5 mM picolyl-azide-PEG4-biotin (Jena Bioscience, CLK-1167-100), 0.1 mM CuSO_4_ (from Click-iT kit), 0.5 mM Tris(3-hydroxypropyltriazolylmethyl)amine (THPTA) (Sigma-Aldrich, 762342), and 10 mM sodium ascorbate (Jena Bioscience, CLK-MI005-1G). DNA fragments between 200 and 700 bp were size-selected using Agencourt AMPure XP beads and resuspended in TE. Next, to capture biotinylated products, MyOne Streptavidin T1 beads (Invitrogen, 65602) were washed three times with 1× BWT buffer [5 mM tris-HCl (pH 7.5), 0.5 mM EDTA, 1 M NaCl, and 0.05% (v/v) Tween 20] and resuspended in 2× BWT buffer at a volume equal to the volume of biotinylated DNA. Streptavidin beads were then mixed with biotinylated DNA and rotated for 30 min at room temperature. Beads containing biotinylated DNA were washed four times with 1× BWT buffer, twice with 1× TE with 0.05% (v/v) Tween 20, and once with 10 mM tris-HCl (pH 7.5). For H3K36me3, H4K20me2 and H3, EdU strands were purified by performing NaOH washes. Last, beads were resuspended in double distilled water. PCR amplification of ChOR-seq samples was performed following the KAPA Hyperprep kit protocol using the streptavidin bead suspension as a template (10 cycles of PCR for IPs and 6 cycles of PCR for inputs). Following PCR, streptavidin beads were purified using a magnetic rack, and the supernatant was cleaned up twice with Agencourt AMPure XP beads at a ratio of 0.8×. Fragment distribution of libraries was checked using the Fragment Analyzer system (Agilent), and libraries were pooled. H3K27me3 samples were sequenced single-end (75 bp) on a NextSeq500 instrument (Illumina). H3K36me3, H4K20me2 and H3 samples were sequenced paired-end on an Illumina NextSeq2000 (Illumina). For direct comparison, dPOLE4 and dPOLE4 MCM2-2A cell lines were assayed together.

#### Data processing

Sequencing reads were adaptor-trimmed using TrimGalore (v0.0.6, Babraham Bioinformatics) and aligned to the mm10 mouse reference genome or the dm6 *drosophila* genome (6.27.1) using bowtie2 (v2.4.269). Only uniquely mapping reads were kept using samtools (v1.1270), and duplicates were removed using Picard MarkDuplicates (v2.26.10, Broad Institute). For read per million (RPM)–based analysis of genome occupancy, bam files were converted to bigwig files using deepTools (v3.5.172) bamCoverage using standard parameters with options–ignoreDuplicates–bl Ensembl mm10 blacklist–e 250 -b 50 and normalized to counts per million (RPM), and replicates were merged with bigWigMerge and visualized using the Integrative Genomics Viewer (IGV) (v2.15.1). To obtain a common H3K27me3 peak dataset, all called peaks (MACS2 v2.2.7.1, --broad) of the individual replicates (*n* = 3) were concatenated and sorted using the BEDtools (v2.30.0,73) SortBed function and overlapping peaks within 500-bp distance merged by the BEDtools Merge function. From this dataset, only peaks that overlap the peak set of all individual replicates were kept using the BEDtools Intersect Interval function with -a specified. The resulting peak sets were used for subsequent analysis. To account for spike-in normalization, samples were normalized to relative reads per million (RRPM) as described in (*1*). For visualization, the RPM-normalized datasets were exported as bigWig files in 50-bp intervals and visualized across selected regions with Seqplots (v1.12.174). To quantify enrichment over peaks, RRPM-normalized datasets in 1-kb window overlapping with common H3K27me3 or H3K36me3 peaks were filtered using Seqmonk. For H3 and H4K20me2, all bins in RRPM-normalized datasets in 1-kb windows were exported. Subsequently, samples were analyzed and visualized in R.

### RNA sequencing

#### Sample preparation

RNA-seq libraries were prepared from POLE4-dTAG cell lines and MCM2-2A/POLE4-dTAG cell lines in triplicate. For serum+LIF conditions, cells were seeded and, after 2 hours, treated with DMSO or dTAG for 24 or 96 hours. For RA differentiation experiments, after 24 hours of dTAG treatment, the media was switched to RA-containing media without LIF, and samples were harvested after 12, 24, and 60 hours of RA exposure. Total RNA was extracted from 5 × 10^6^ cells using the RNeasy Plus Mini Kit (QIAGEN) according to the manufacturer’s protocol, and gDNA was eliminated by treatment with the RNase-Free DNase Set (QIAGEN). Quality of RNA was assessed using a 2100 Bioanalyzer RNA 6000 Nano kit (Agilent). Total RNA (500 ng) from each sample was depleted of ribosomal RNA (rRNA) using the NEBNext rRNA Depletion kit (NEB). Strand-specific RNA libraries were prepared using the NEBNext Ultra Directional RNA Library Prep kit (NEB), assessed on the Bioanalyzer High Sensitivity DNA kit (Agilent) to ensure good quality, and sequenced as 100-bp paired-end reads on the Illumina NextSeq 2000 platform in three biological replicates. For direct comparison, dPOLE4 and dPOLE4 MCM2-2A cell lines were assayed together.

#### Data processing

Reads were quality trimmed and filtered for adapters with cutadapt (version 4.2) and mapped to the mouse genome (mm10) using STAR mapper (version 2.7.11a) with special multimapping parameters --winAnchorMultimapNmax 200 and --outFilterMultimapNmax. Quantification of genes and repeat subfamilies was performed with the TEcount tool, part of the TETranscripts (version 2.2.3) software suite using gencode (version M25) annotations and TETranscripts custom repeat annotations GRCm38_GENCODE_rmsk_TE. Differential expression analysis was performed with Deseq2. DE genes/repeat subfamilies were defined as those with |fold change| > 1.5 and false discover rate (FDR) < 0.01. Gene Ontology term functional enrichment was performed with the package clusterProfiler18 (version 3.18.1).

### Replication transposase-accessible chromatin with sequencing

#### Sample preparation

A detailed step-by-step protocol is available (*65*). To generate nascent repli-ATAC-seq samples, we used MCM2-2A/PolE4-dTAG cells in two biological replicates. Cells were subjected to DMSO or dTAG-13 treatment for 2 hours, followed by pulsing in EdU-containing media (10 μM) for 10 min. Nascent samples were prepared by trypsinizing the cells (Gibco, 25200056). After cell counting, 100,000 cells were used for repli-ATAC followed by nuclei isolation. The nuclei were treated with Tagment DNA Enzyme (TDE1) (Illumina, 15027865) for 30 min, and the tagmented DNA was purified. EdU-labeled DNA was biotinylated using Click-IT and purified with Dynabeads MyOne Streptavidin T1 beads (Invitrogen, 65602). The purified tagmented DNA was subjected to size selection up to 1000 bp using a double-size selection method with Agencourt AMPure XP beads (Beckman Coulter, A63881). To isolate biotinylated fragments, 20 μl of Dynabeads MyOne Streptavidin T1 beads (Invitrogen, 65602) per sample was washed three times with 1× B&W buffer [5 mM tris-HCl (pH 7.5,) 0.5 mM EDTA, 1 M NaCl, and 0.05% Tween 20] and resuspended in 2× B&W buffer at a volume equal to the volume of biotinylated DNA. After adding the streptavidin beads to the DNA sample, samples were incubated for 30 min at room temperature with side rotation. Next, beads were then pelleted on a magnetic rack and resuspended in water. Repli-ATAC-seq libraries were PCR-amplified with the following reaction mix: 50 nM primer 1, 50 nM indexed primer 2, and 1× NEBNext High-Fidelity PCR Master Mix (NEB, M0541). Libraries were amplified with the following conditions: 72°C, 5 min; 98°C, 30 s; 12 repli-ATAC-seq libraries cycles of 98°C, 10 s; 63°C, 30 s; 72°C, 30 s. Libraries were purified twice using a 1.6:1 AMPure XP bead ratio and resuspended in 10 μl of elution buffer (EB). Fragment distribution of libraries was checked using the Fragment Analyzer system (Agilent), and libraries were pooled and sequenced paired end (68 bp) on a NextSeq2000 instrument (Illumina).

#### Data processing

Samples were processed and quality controlled using the Encyclopedia of DNA Elements (ENCODE) standardized pipeline for ATAC-seq (v2.2.2). Briefly, adapters were removed, reads were trimmed with cutadapt, mapping was performed with bowtie2 to a hybrid mm10 and dm6 genomes, bam files were filtered, using only reads with mapping quality (MAPQ) ≥ 30, and reads falling in mm10 encode blacklisted regions were removed. The bam files were down-sampled to the same size as the lowest sequences library using Picard (version 2.27.5). Down-sample bamfiles were merged, and bigwig files for all repli-ATAC samples were computed using deepTools bamcoverage (version 3.5.1) in bins of 50 bp and normalized using Reads Per Kilobase per Million mapped reads (RPKM). Bigwig files were processed with deepTools multibigwigsummary. Enrichment plots around active TSSs were made by deepTools plotProfile.

### Statistical analysis

The statistical test was performed using GraphPad Prism and R software. The statistics for the high-content microscopy experiments was performed on the mean of individual replicates.
