# Supplementary material for: Disabling leading and lagging strand histone transmission results in parental histones loss and reduced cell plasticity and viability
Source: Sci Adv. 2025 Feb 19;11(8):eadr1453. doi: 10.1126/sciadv.adr1453 (PMC11837984; doi:10.1126/sciadv.adr1453)
Supplement: Supplementary file 1 — Figs. S1 to S4 Table S1 [file sciadv.adr1453_sm.pdf]

Science Advances
AAAS

Supplementary Materials for

Disabling leading and lagging strand histone transmission results in parental histones loss and reduced cell plasticity and viability

Leonie Kollenstart *et al.*

Corresponding author: Anja Groth, anja.groth@cpr.ku.dk

Sci. Adv. **11**, eadr1453 (2025)
DOI: 10.1126/sciadv.adr1453

This PDF file includes:

Figs. S1 to S4
Table S1

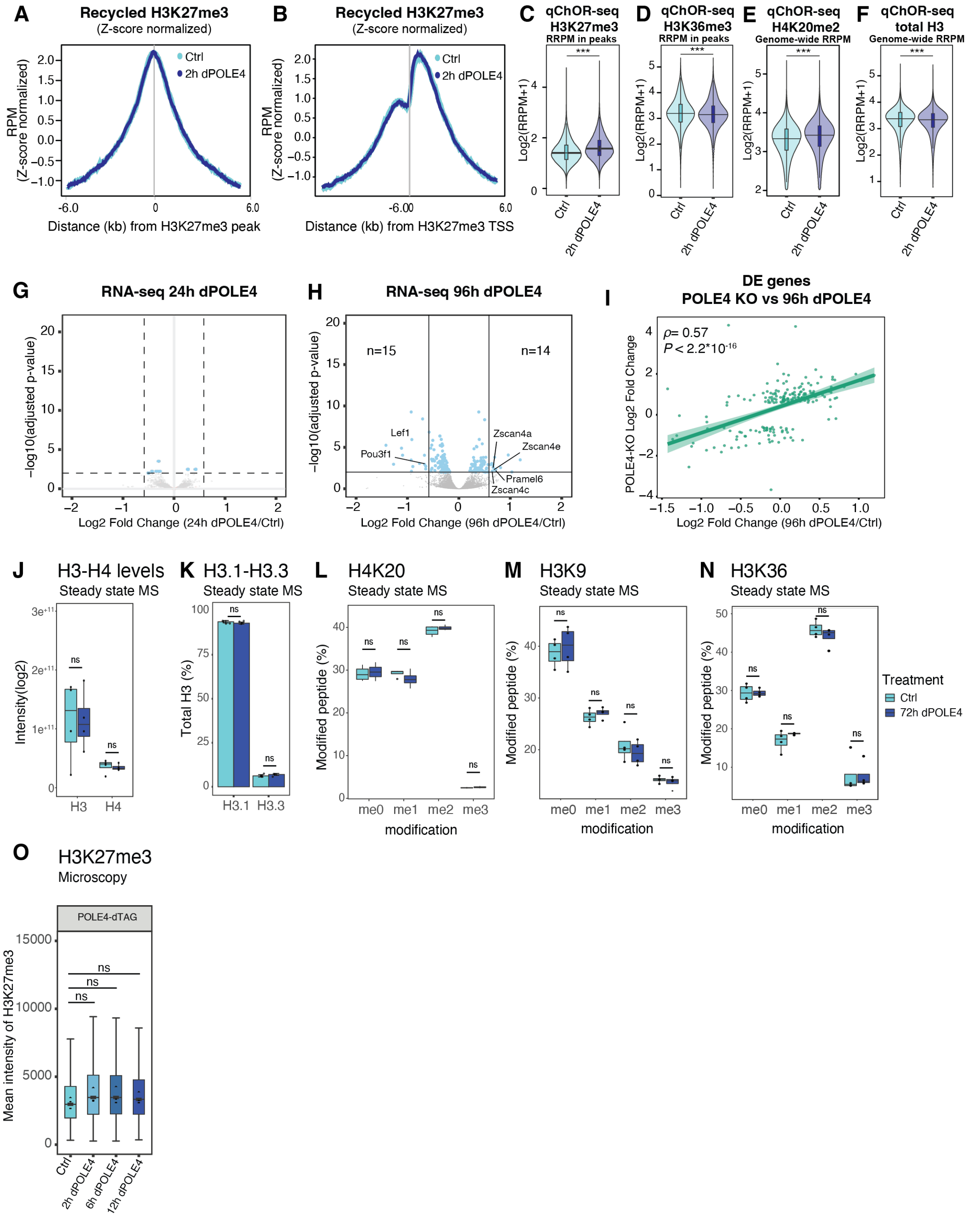

Supplemental Figure 1. Effects of POLE4 depletion on H3K27me3 occupancy, transcription and H3-H4 (hPTMs).

(A) Average profile of nascent H3K27me3 occupancy across total H3K27me3-peaks in DMSO (Ctrl, light blue) or dTAG-13 (dPOLE4, dark blue) treated POLE4-dTAG mESCs. RPM scale, Z-score normalized. Average of two independent replicates. (**B**) Average profile of nascent H3K27me3 occupancy across transcription start sites (TSSs) in DMSO (Ctrl, light blue) or dTAG-13 (dPOLE4, dark blue) treated POLE4-dTAG mESCs. RPM scale, Z-score normalized. Average of two independent

replicates. (**C-F**) quantitative ChOR-seq signal in 1 kb bins of recycled (**C**) H3K27me3 and **(D)** H3K36me3 overlapping total peaks or (**E**) genome-wide H4K20me2 and (**F**) total H3 in DMSO or 2h dTAG-13. Signal quantified with reference-adjusted reads per million using exogenous spike-in chromatin (RRPM). Wilcoxon signed-rank test. n = 3 biological replicates. Note that recycling accuracy cannot be estimated using H4K20me2, as this mark is deposited on approximately 40% of histones genome wide (L). **(G)** Volcano plot showing differential expression analysis of genes and repeat subfamily expression. Fold change against false discovery rate (FDR) adjusted P-value is shown per gene in 24 hours dTAG-13 (dPOLE4) versus DMSO (Ctrl) in POLE4-dTAG cells. n = 3 biological replicates. **(H)** Volcano plot showing differential expression analysis of genes and repeat subfamily expression. Fold change against false discovery rate (FDR) adjusted P-value is shown per gene after 96 hours dTAG-13 (dPOLE4) versus DMSO (Ctrl) treatment of POLE4-dTAG mESCs. n = 3 biological replicates (**(I)** Scatter plot showing gene expression changes at 96 hours dTAG (dPOLE4) treatment of POLE4-dTAG mESC RNA-seq (H) against POLE4-KO mESC (Wenger et al. 2023) log2FC > 0.58. All DE genes in either POLE4-dTAG or POLE4-KO cells are plotted. Spearman's rank correlation coefficient (ρ) with P-value. **(J)** Box plots showing total histone levels quantified by mass spectrometry (n = 4). (**K)** Box plots showing H3.1 and H3.3 relative to total H3 quantified by mass spectrometry (n = 4). **(L)** Box plots showing global H4K20 methylation levels quantified by mass spectrometry (n=4). **(M)** Box plots showing global H3K9 methylation levels in POLE4-dTAG cells quantified by mass spectrometry (n=4). **(N)** Box plots showing global H3K36me3 methylation levels in POLE4-dTAG cells quantified by mass spectrometry (n=4). (**O**) H3K27me3 levels quantified with high-content microscopy in POLE4-dTAG mESC treated for 2, 4, 6, and 12 hours with DMSO (Ctrl) or dTAG-13 (dPOLE4). n = 3 biological replicates. P values, Student t test (two-tailed, paired), performed on the means of the biological replicates.

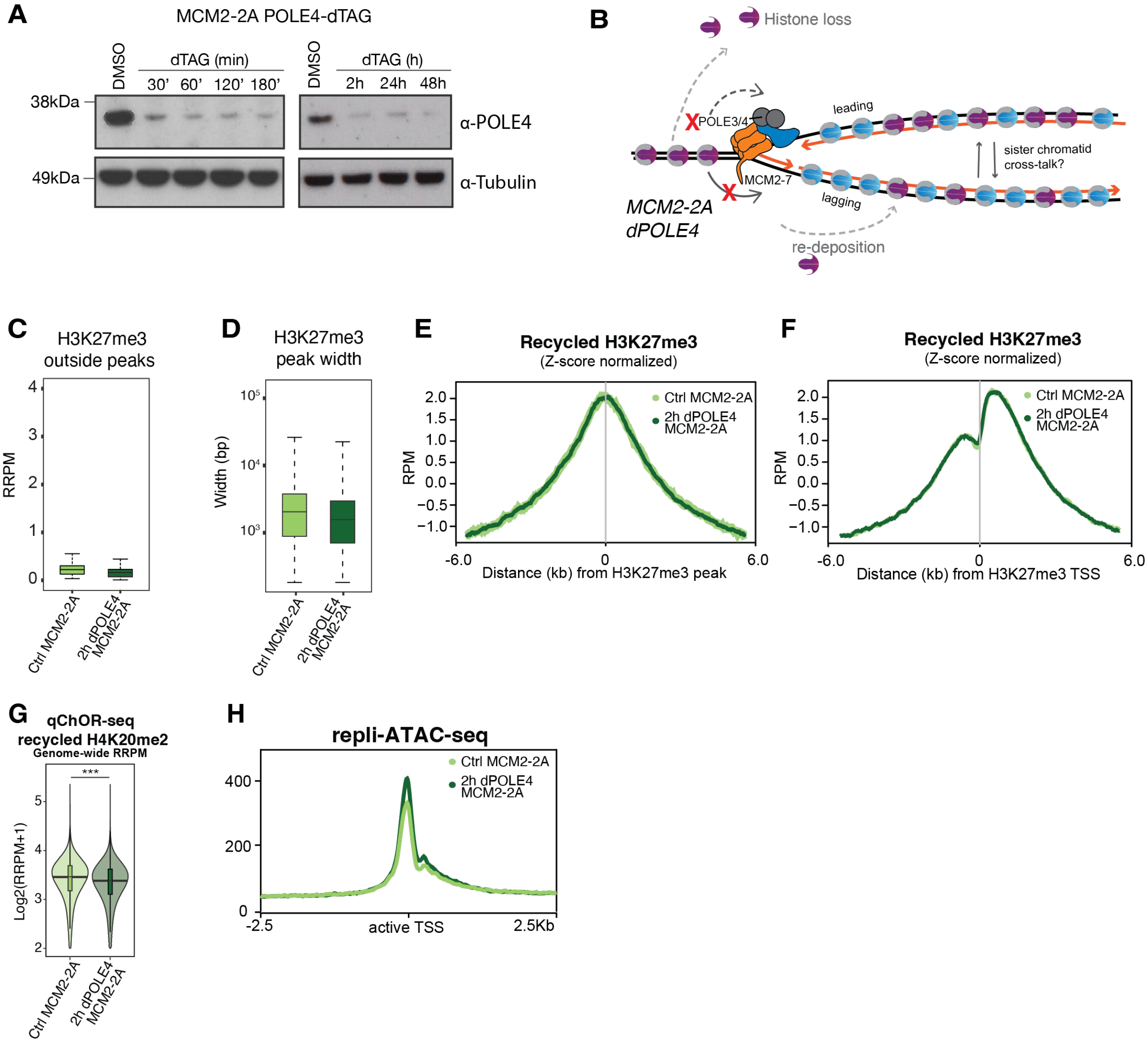

Supplemental Figure 2. POLE4 depletion in MCM2-2A cells decreases H3K27me3 levels but does not affect relative H3K27me3 distribution.

(A) Western blot analysis of POLE4-dTAG MCM2-2A mESCs treated with DMSO (Ctrl MCM2-2A) and dTAG-13 (dPOLE4 MCM2-2A). **(B)** Model of histone recycling upon removal of POLE4 in an MCM2-2A background. Recycling is ongoing to both strands with a preference to the leading strand. Possibility of an increase of parental histones on the lagging strand and/or a decrease of parental histones on the leading strand compared to the single MCM2-2A background. **(C)** ChOR-seq signal of recycled H3K27me3 in 1 kb windows not overlapping the total H3K27me3 peaks upon DMSO (Ctrl MCM2-2A) or dTAG-13 (dPOLE4 MCM2-2A) treatment. Log2(RRPM + 1) scale. Black line, median; dashed lines, 1.5× interquartile range. **(D)** Peak width (bp) of nascent H3K27me3 (ChOR-seq) calculated with MACS2 upon DMSO (Ctrl MCM2-2A) or dTAG-13 (dPOLE4 MCM2-2A) treatment. Black line, median; dashed lines, 1.5× interquartile range. **(E)** Average profile of recycled H3K27me3 occupancy across total H3K27me3-peaks in DMSO (Ctrl MCM2-2A, light green) or dTAG-13 (dPOLE4 MCM2-2A, dark green) treated POLE4-dTAG MCM2-2A mESCs. RPM scale, Z-score normalized. n = 2 biological replicates**. (F)** Average profile of recycled H3K27me3 occupancy across H3K27me3-containing TSSs in DMSO (Ctrl MCM2-2A, light green) or dTAG-13 (dPOLE4 MCM2-2A, dark green) treated POLE4-

dTAG MCM2-2A cells. RPM scale, Z-score normalized. (n = 2 biological replicates)**. (G)** ChOR-seq signal of recycled H4K20me2 in 1kb bins genome-wide in DMSO (Ctrl MCM2-2A, light green) or dTAG-13 (dPOLE4 MCM2-2A, dark green) treated POLE4-dTAG MCM2-2A cells. Analyzed as in 2F. n = 3 biological replicates. **(H)** Average profile of nascent repli-ATAC-seq signal over 5 kb regions centered on the TSSs of active genes in POLE4-dTAG MCM2-2A mESCs.

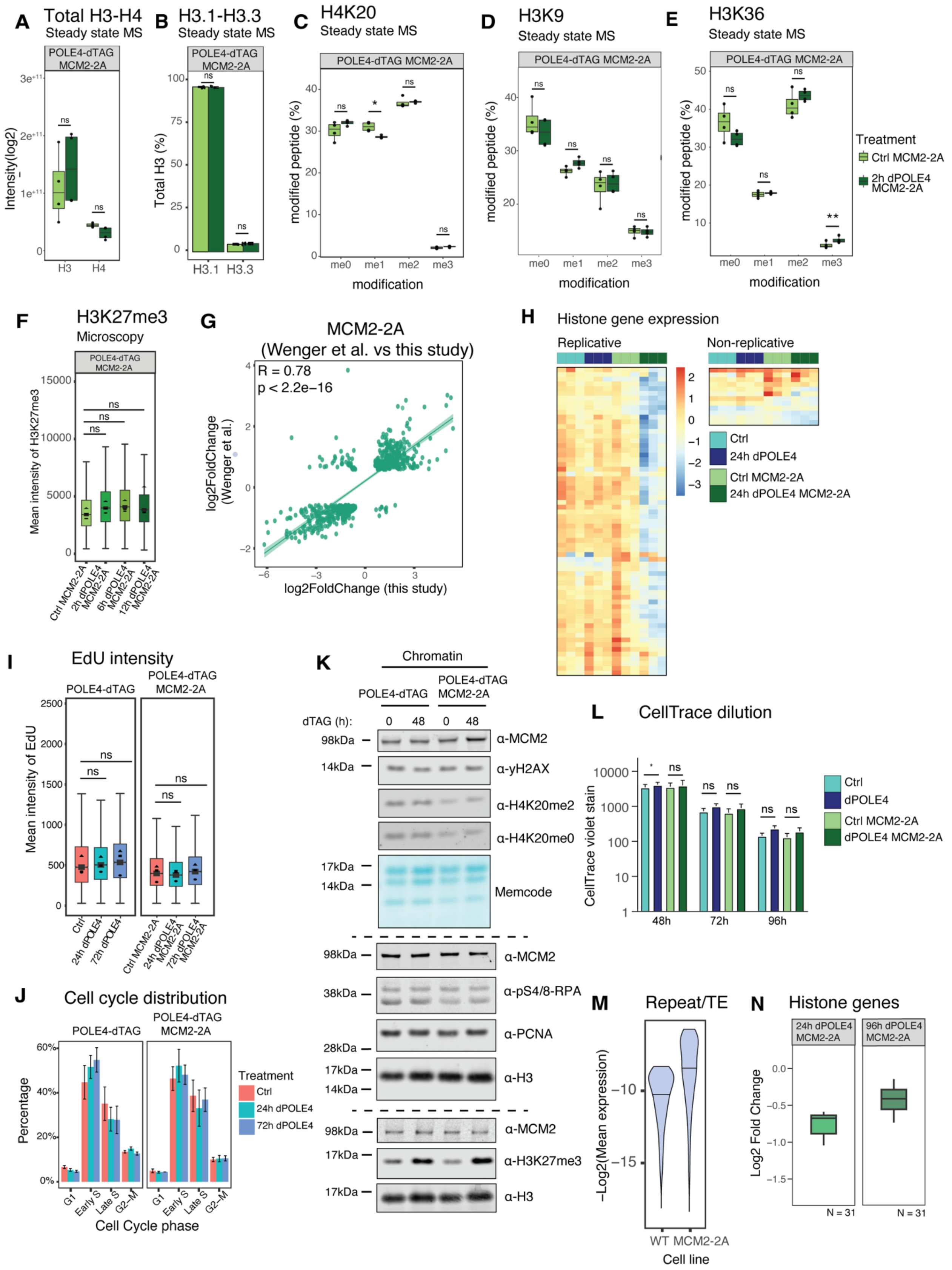

A Total H3-H4 Steady state MS
POLE4-dTAG MCM2-2A
Intensity(log2)
H3
H4
B H3.1-H3.3 Steady state MS
Total H3 (%)
H3.1
H3.3
C H4K20 Steady state MS
POLE4-dTAG MCM2-2A
modified peptide (%)
modification
D H3K9 Steady state MS
E H3K36 Steady state MS
Treatment
Ctrl MCM2-2A
2h dPOLE4 MCM2-2A
F H3K27me3 Microscopy
Mean intensity of H3K27me3
Ctrl MCM2-2A
2h dPOLE4 MCM2-2A
6h dPOLE4 MCM2-2A
12h dPOLE4 MCM2-2A
G MCM2-2A (Wenger et al. vs this study)
R = 0.78
p < 2.2e−16
log2FoldChange (Wenger et al.)
log2FoldChange (this study)
H Histone gene expression
Replicative
Non-replicative
Ctrl
24h dPOLE4
Ctrl MCM2-2A
24h dPOLE4 MCM2-2A
I EdU intensity
POLE4-dTAG
POLE4-dTAG MCM2-2A
Mean intensity of EdU
Ctrl
24h dPOLE4
72h dPOLE4
Ctrl MCM2-2A
24h dPOLE4 MCM2-2A
72h dPOLE4 MCM2-2A
J Cell cycle distribution
Percentage
Treatment
Ctrl
24h dPOLE4
72h dPOLE4
G1
Early S
Late S
G2–M
Cell Cycle phase
K Chromatin
dTAG (h):
α-MCM2
α-γH2AX
α-H4K20me2
α-H4K20me0
Memcode
α-pS4/8-RPA
α-PCNA
α-H3
α-H3K27me3
L CellTrace dilution
CellTrace violet stain
Ctrl
dPOLE4
Ctrl MCM2-2A
dPOLE4 MCM2-2A
48h
72h
96h
M Repeat/TE
−Log2(Mean expression)
WT
MCM2-2A
Cell line
N Histone genes
24h dPOLE4 MCM2-2A
96h dPOLE4 MCM2-2A
Log2 Fold Change
N = 31

Supplemental Figure 3. The effect of POLE4 depletion in MCM2-2A cells on H3-H4 (hPTMs), repeat expression, cell cycle progression and DNA damage.
(A) Box plots showing total histone levels in DMSO (Ctrl MCM2-2A) and 72h dTAG-13 (dPOLE4 MCM2-2A) POLE4-dTAG MCM2-2A mESCs quantified by mass spectrometry using a H3 and a H4 peptide that does not contain sites for PTMs. n = 4 biological replicates. **(B)** Bar plot showing H3.1 and H3.3 relative to total H3 POLE4-dTAG MCM2-2A mESCs quantified by mass spectrometry (n = 4). **(C-F)** Box plots showing global H4K20 methylation levels POLE4-dTAG MCM2-2A mESCs quantified by mass spectrometry. n=4 biological replicates. **(D)** Box plots showing global H3K9 methylation levels in POLE4-dTAG MCM2-2A mESCs quantified by mass spectrometry (n=4 biological replicates). **(E)** Box plots showing global H3K36me3 methylation levels in POLE4-dTAG MCM2-2A mESCs quantified by mass spectrometry (n=4 biological replicates). **(F)** H3K27me3 levels quantified with high-content microscopy in POLE4-dTAG MCM2-2A mESCs treated for 2, 4, 6, or 12 hours with DMSO (Ctrl) or dTAG-13 (dPOLE4). n = 3 biological replicates. P values, Student t test (two-tailed, paired), performed on the means of the biological replicates. **(G)** Scatter plot showing expression changes in of MCM2-2A mESCs RNA-seq (Ctrl MCM2-2A/Ctrl, this study) compared to MCM2-2A mESCs RNA-seq data (Wenger et al. 2023) log2FC > 0.58. Spearman's rank correlation coefficient (ρ) with P-value. **(H)** Heatmaps of Zscore normalized histone transcription levels in POLE4-dTAG and MCM2-2A POLE4-dTAG mESCs treated for 24 hours with DMSO (Ctrl, Ctrl MCM2-2A) or dTAG (dPOLE4, dPOLE4 MCM2-2A). Each column represents an individual biological replicate (R1, R2, R3). **(I)** High-content microscopy measurement of mean EdU intensity in POLE4-dTAG and POLE4-dTAG MCM2-2A mESCs pulsed with EdU and treated for 72 hours with DMSO (Ctrl, Ctrl MCM2-2A) or dTAG-13 (dPOLE4, dPOLE4 MCM2-2A). n = 3 biological replicates. **(J)** High-content microscopy analysis of cell cycle progression in POLE4-dTAG and POLE4-dTAG MCM2-2A mESCs pulsed with EdU and treated for 72 hours with DMSO (Ctrl, Ctrl MCM2-2A) or dTAG-13 (dPOLE4, dPOLE4 MCM2-2A). Cell-cycle stages were defined by gating on mean EdU intensity and total DAPI intensity. Mean ± s.d. intensities are shown as percentage of total fraction. n = 3 biological replicates. **(K)** Western blot analysis of chromatin fractionation of POLE4-dTAG and POLE4-dTAG MCM2-2A mESCs treated for 48 hours with DMSO (Ctrl, Ctrl MCM2-2A) or dTAG-13 (dPOLE4, dPOLE4 MCM2-2A) with indicated antibodies. **(L)** Cell Trace division assay, cells were grown in CellTrace Violet for the indicated times and treatment. n = 3 biological replicates. **(M)** BaseMean (average of the normalized count values, divided by size factors) for WT (DMSO, Ctrl) and MCM2-2A (DMSO, Ctrl MCM2-2A) showing repeat and transposable element expression. **(N)** Log2 fold changes in histone gene expression for short (24 hours) and long (96 hours) time courses of POLE4 depletion in POLE4-dTAG MCM2-2A mESCs.

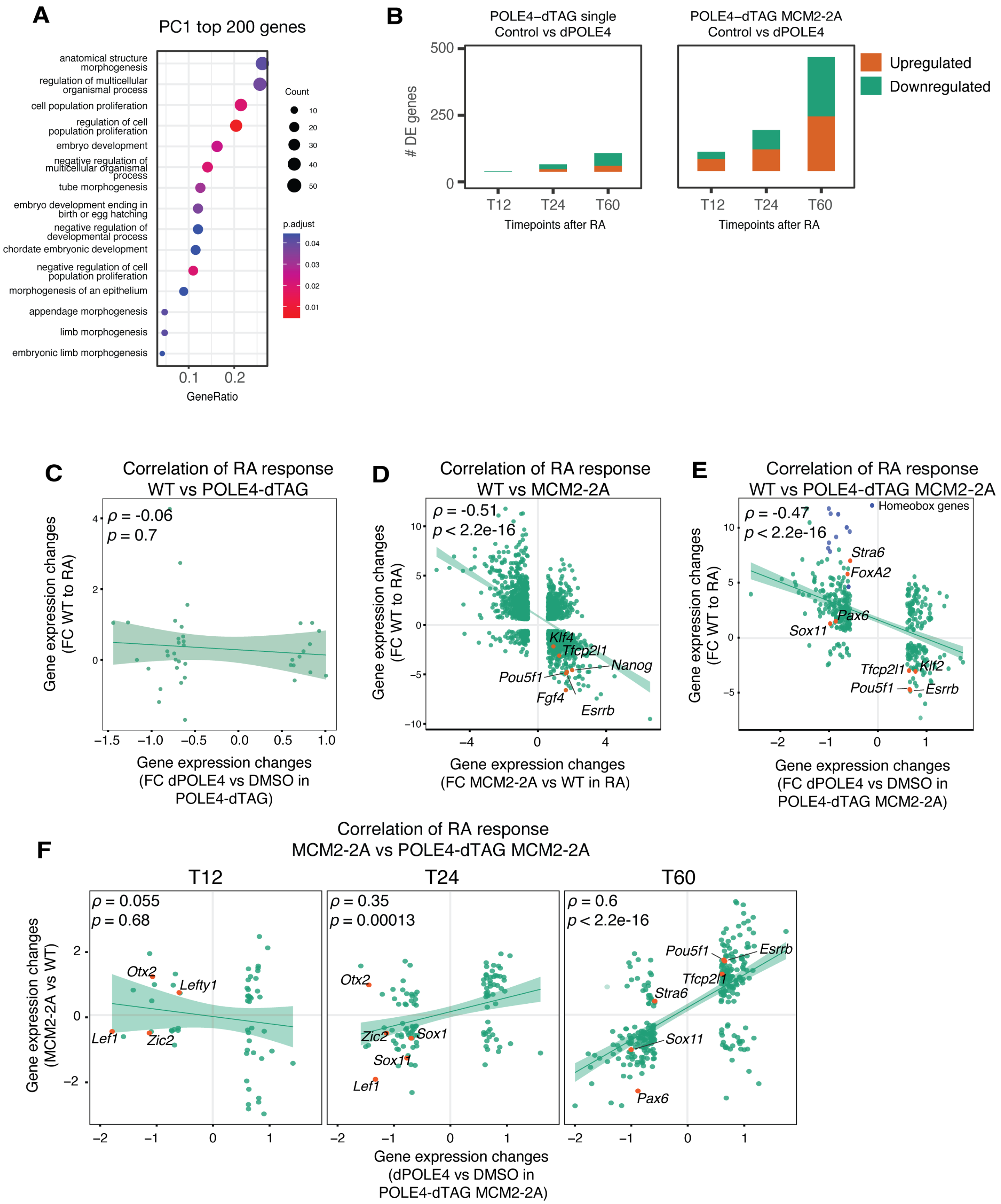

Supplemental Figure 4 Transcriptional response to POLE4 depletion in MCM2-2A cells during retinoic acid (RA) differentiation.

(A) Dot plot of GO term enrichment analysis for biological processes using the top 100 genes found in in PC1 (see Fig. 4B) from RNA-seq data during RA treatment. **(B)** Number differentially expressed genes found after RA treatment (excluding DE genes at T0). Comparing dTAG (dPOLE4) versus DMSO (Ctrl)

for POLE4-dTAG cells and dTAG (dPOLE4 MCM2-2A) versus DMSO (Ctrl MCM2-2A) for POLE4-dTAG MCM2-2A mESC. **(C)** Scatter plots showing the expression changes (log2 FC) of DE genes between 60 hours RA treatment versus untreated wild type ((Ctrl T60/Ctrl T0) on the y-axis and expression changes in POLE4 depleted cells (dPOLE4 T60/Ctrl T60) on the x-axis. | log2 FC | > 0.58, adjusted P-value < 0.01. Spearman's rank correlation coefficient (ρ) with P-value. **(D)** Scatter plots showing the expression changes (log2 FC) of DE genes between 60 hours RA treatment versus untreated wild type (Ctrl T60/Ctrl T0) on the y-axis and expression changes at 60 hours RA treatment in MCM2-2A single mutants (Ctrl MCM2-2A/Ctrl) on the x-axis. DE genes defined by | log2 FC | > 0.58, adjusted P-value < 0.01). Spearman's rank correlation coefficient (ρ) with P-value. **(E)** Scatter plots showing the expression changes (log2 FC) of DE genes between 60 hours retinoic acid vs untreated wild type (Ctrl T60/Ctrl T0) on the y-axis and DE genes at 60 hours retinoic acid in POLE4-dTAG MCM2-2A POLE4 depleted cells (dPOLE4 MCM2-2A T60/Ctrl MCM2-2A T60) on the x-axis. DE genes defined by | log2 FC | > 0.58, adjusted P-value < 0.01). Spearman's rank correlation coefficient (ρ) with P-value. (**F**) Scatter plots showing correlation of fold changes for DE genes at RA differentiation time points (T12, T24, and T60) between single MCM2-2A (Ctrl MCM2-2A/Ctrl) on the y-axis and DE genes in MCM2-2A POLE4 depleted cells (dPOLE4 MCM2-2A/Ctrl MCM2-2A) on the x-axis. DE genes defined by | log2 FC | > 0.58, adjusted P-value < 0.01). Spearman's rank correlation coefficient (ρ) with P-value. (**A-F**) n = 3 biological replicates.

Gene name	log2FC	padj	Ensemble gene ID	background	comparison	dPOLE4
Ccne1	0.151	0.055	ENSMUSG00000002068	MCM2_2A	treatment_dTAG_vs_DMSO	T96
Cdk4	-0.022	0.947	ENSMUSG00000006728	MCM2_2A	treatment_dTAG_vs_DMSO	T96
Ccn2	0.218	0.202	ENSMUSG00000019997	MCM2_2A	treatment_dTAG_vs_DMSO	T96
Mdm2	0.109	0.466	ENSMUSG00000020184	MCM2_2A	treatment_dTAG_vs_DMSO	T96
Cdc34	-0.062	0.778	ENSMUSG00000020307	MCM2_2A	treatment_dTAG_vs_DMSO	T96
Pold2	-0.030	0.959	ENSMUSG00000020471	MCM2_2A	treatment_dTAG_vs_DMSO	T96
Cdkn1a	0.204	0.158	ENSMUSG00000023067	MCM2_2A	treatment_dTAG_vs_DMSO	T96
Pcbp4	-0.294	0.004	ENSMUSG00000023495	MCM2_2A	treatment_dTAG_vs_DMSO	T96
Cdk2	0.062	0.884	ENSMUSG00000025358	MCM2_2A	treatment_dTAG_vs_DMSO	T96
Mcm6	-0.083	0.453	ENSMUSG00000026355	MCM2_2A	treatment_dTAG_vs_DMSO	T96
Pcna	0.026	0.938	ENSMUSG00000027342	MCM2_2A	treatment_dTAG_vs_DMSO	T96
Ccna2	0.043	0.897	ENSMUSG00000027715	MCM2_2A	treatment_dTAG_vs_DMSO	T96
Plk1	-0.114	0.382	ENSMUSG00000030867	MCM2_2A	treatment_dTAG_vs_DMSO	T96
Gas6	0.356	0.249	ENSMUSG00000031451	MCM2_2A	treatment_dTAG_vs_DMSO	T96
Casp3	-0.052	0.941	ENSMUSG00000031628	MCM2_2A	treatment_dTAG_vs_DMSO	T96
Cenpn	-0.057	0.938	ENSMUSG00000031756	MCM2_2A	treatment_dTAG_vs_DMSO	T96
Cdkn1c	0.315	0.255	ENSMUSG00000037664	MCM2_2A	treatment_dTAG_vs_DMSO	T96
Dap	-0.180	0.880	ENSMUSG00000039168	MCM2_2A	treatment_dTAG_vs_DMSO	T96
Ccnb1	-0.073	0.617	ENSMUSG00000041431	MCM2_2A	treatment_dTAG_vs_DMSO	T96
Mcm3	-0.107	0.188	ENSMUSG00000041859	MCM2_2A	treatment_dTAG_vs_DMSO	T96
Gas1	-0.177	NA	ENSMUSG00000052957	MCM2_2A	treatment_dTAG_vs_DMSO	T96
Trp53	-0.092	0.350	ENSMUSG00000059552	MCM2_2A	treatment_dTAG_vs_DMSO	T96
Ccnd1	-0.222	0.012	ENSMUSG00000070348	MCM2_2A	treatment_dTAG_vs_DMSO	T96
Ccne1	0.123	0.275	ENSMUSG00000002068	WT	treatment_dTAG_vs_DMSO	T96
Cdk4	-0.010	0.973	ENSMUSG00000006728	WT	treatment_dTAG_vs_DMSO	T96
Ccn2	-0.065	0.815	ENSMUSG00000019997	WT	treatment_dTAG_vs_DMSO	T96
Mdm2	0.194	0.044	ENSMUSG00000020184	WT	treatment_dTAG_vs_DMSO	T96
Cdc34	0.029	0.910	ENSMUSG00000020307	WT	treatment_dTAG_vs_DMSO	T96
Pold2	0.043	0.884	ENSMUSG00000020471	WT	treatment_dTAG_vs_DMSO	T96
Cdkn1a	0.275	0.046	ENSMUSG00000023067	WT	treatment_dTAG_vs_DMSO	T96
Pcbp4	-0.106	0.559	ENSMUSG00000023495	WT	treatment_dTAG_vs_DMSO	T96

Cdk2	0.036	0.915	ENSMUSG00000025358	WT	treatment_dTAG_vs_DMSO	T96
Mcm6	-0.079	0.577	ENSMUSG00000026355	WT	treatment_dTAG_vs_DMSO	T96
Pcna	0.048	0.815	ENSMUSG00000027342	WT	treatment_dTAG_vs_DMSO	T96
Ccna2	-0.014	0.961	ENSMUSG00000027715	WT	treatment_dTAG_vs_DMSO	T96
Plk1	-0.026	0.931	ENSMUSG00000030867	WT	treatment_dTAG_vs_DMSO	T96
Gas6	0.386	0.232	ENSMUSG00000031451	WT	treatment_dTAG_vs_DMSO	T96
Casp3	-0.128	0.692	ENSMUSG00000031628	WT	treatment_dTAG_vs_DMSO	T96
Cenpn	-0.005	0.992	ENSMUSG00000031756	WT	treatment_dTAG_vs_DMSO	T96
Cdkn1c	-0.225	0.524	ENSMUSG00000037664	WT	treatment_dTAG_vs_DMSO	T96
Dap	0.275	0.752	ENSMUSG00000039168	WT	treatment_dTAG_vs_DMSO	T96
Ccnb1	-0.164	0.067	ENSMUSG00000041431	WT	treatment_dTAG_vs_DMSO	T96
Mcm3	-0.028	0.904	ENSMUSG00000041859	WT	treatment_dTAG_vs_DMSO	T96
Gas1	-0.307	NA	ENSMUSG00000052957	WT	treatment_dTAG_vs_DMSO	T96
Trp53	-0.028	0.893	ENSMUSG00000059552	WT	treatment_dTAG_vs_DMSO	T96
Ccnd1	-0.105	0.524	ENSMUSG00000070348	WT	treatment_dTAG_vs_DMSO	T96
Ccne1	0.081	0.577	ENSMUSG00000002068	MCM2_2A	treatment_dTAG_vs_DMSO	T24
Cdk4	-0.082	0.539	ENSMUSG00000006728	MCM2_2A	treatment_dTAG_vs_DMSO	T24
Ccn2	0.055	0.894	ENSMUSG00000019997	MCM2_2A	treatment_dTAG_vs_DMSO	T24
Mdm2	0.381	0.000	ENSMUSG00000020184	MCM2_2A	treatment_dTAG_vs_DMSO	T24
Cdc34	-0.121	0.279	ENSMUSG00000020307	MCM2_2A	treatment_dTAG_vs_DMSO	T24
Pold2	-0.066	0.755	ENSMUSG00000020471	MCM2_2A	treatment_dTAG_vs_DMSO	T24
Cdkn1a	0.266	0.066	ENSMUSG00000023067	MCM2_2A	treatment_dTAG_vs_DMSO	T24
Pcbp4	-0.119	0.426	ENSMUSG00000023495	MCM2_2A	treatment_dTAG_vs_DMSO	T24
Cdk2	0.004	0.989	ENSMUSG00000025358	MCM2_2A	treatment_dTAG_vs_DMSO	T24
Mcm6	-0.120	0.267	ENSMUSG00000026355	MCM2_2A	treatment_dTAG_vs_DMSO	T24
Pcna	0.061	0.698	ENSMUSG00000027342	MCM2_2A	treatment_dTAG_vs_DMSO	T24
Ccna2	-0.141	0.218	ENSMUSG00000027715	MCM2_2A	treatment_dTAG_vs_DMSO	T24
Plk1	-0.291	0.000	ENSMUSG00000030867	MCM2_2A	treatment_dTAG_vs_DMSO	T24
Gas6	0.335	0.256	ENSMUSG00000031451	MCM2_2A	treatment_dTAG_vs_DMSO	T24
Casp3	0.039	0.915	ENSMUSG00000031628	MCM2_2A	treatment_dTAG_vs_DMSO	T24
Cenpn	-0.011	0.983	ENSMUSG00000031756	MCM2_2A	treatment_dTAG_vs_DMSO	T24
Cdkn1c	0.110	0.779	ENSMUSG00000037664	MCM2_2A	treatment_dTAG_vs_DMSO	T24
Dap	-0.188	0.741	ENSMUSG00000039168	MCM2_2A	treatment_dTAG_vs_DMSO	T24

Ccnb1	-0.164	0.094	ENSMUSG00000041431	MCM2_2A	treatment_dTAG_vs_DMSO	T24
Mcm3	-0.079	0.553	ENSMUSG00000041859	MCM2_2A	treatment_dTAG_vs_DMSO	T24
Gas1	0.016	NA	ENSMUSG00000052957	MCM2_2A	treatment_dTAG_vs_DMSO	T24
Trp53	-0.200	0.041	ENSMUSG00000059552	MCM2_2A	treatment_dTAG_vs_DMSO	T24
Ccnd1	-0.135	0.279	ENSMUSG00000070348	MCM2_2A	treatment_dTAG_vs_DMSO	T24
Ccne1	0.140	0.152	ENSMUSG00000002068	WT	treatment_dTAG_vs_DMSO	T24
Cdk4	0.057	0.573	ENSMUSG00000006728	WT	treatment_dTAG_vs_DMSO	T24
Ccn2	0.132	0.298	ENSMUSG00000019997	WT	treatment_dTAG_vs_DMSO	T24
Mdm2	0.160	0.118	ENSMUSG00000020184	WT	treatment_dTAG_vs_DMSO	T24
Cdc34	0.140	0.148	ENSMUSG00000020307	WT	treatment_dTAG_vs_DMSO	T24
Pold2	0.034	0.818	ENSMUSG00000020471	WT	treatment_dTAG_vs_DMSO	T24
Cdkn1a	0.131	NA	ENSMUSG00000023067	WT	treatment_dTAG_vs_DMSO	T24
Pcbp4	0.077	0.603	ENSMUSG00000023495	WT	treatment_dTAG_vs_DMSO	T24
Cdk2	0.100	0.460	ENSMUSG00000025358	WT	treatment_dTAG_vs_DMSO	T24
Mcm6	-0.015	0.915	ENSMUSG00000026355	WT	treatment_dTAG_vs_DMSO	T24
Pcna	0.134	0.138	ENSMUSG00000027342	WT	treatment_dTAG_vs_DMSO	T24
Ccna2	-0.028	0.837	ENSMUSG00000027715	WT	treatment_dTAG_vs_DMSO	T24
Plk1	0.062	0.620	ENSMUSG00000030867	WT	treatment_dTAG_vs_DMSO	T24
Gas6	0.129	NA	ENSMUSG00000031451	WT	treatment_dTAG_vs_DMSO	T24
Casp3	0.089	NA	ENSMUSG00000031628	WT	treatment_dTAG_vs_DMSO	T24
Cenpn	-0.040	NA	ENSMUSG00000031756	WT	treatment_dTAG_vs_DMSO	T24
Cdkn1c	0.100	NA	ENSMUSG00000037664	WT	treatment_dTAG_vs_DMSO	T24
Dap	0.237	NA	ENSMUSG00000039168	WT	treatment_dTAG_vs_DMSO	T24
Ccnb1	-0.082	0.462	ENSMUSG00000041431	WT	treatment_dTAG_vs_DMSO	T24
Mcm3	0.049	0.623	ENSMUSG00000041859	WT	treatment_dTAG_vs_DMSO	T24
Gas1	-0.170	NA	ENSMUSG00000052957	WT	treatment_dTAG_vs_DMSO	T24
Trp53	-0.056	0.557	ENSMUSG00000059552	WT	treatment_dTAG_vs_DMSO	T24
Ccnd1	0.057	0.655	ENSMUSG00000070348	WT	treatment_dTAG_vs_DMSO	T24

Table S1 Transcriptional effects cell cycle genes

Log2 Fold Change (FC) of cell cycle genes in WT and MCM2-2A background after 24 hours or 96 hours of dPOLE4.
